# Material Stiffness in Cooperation with Macrophage Paracrine Signals Determines the Tenogenic Differentiation of Mesenchymal Stem Cells

**DOI:** 10.1002/advs.202206814

**Published:** 2023-04-25

**Authors:** Renwang Sheng, Jia Liu, Wei Zhang, Yifan Luo, Zhixuan Chen, Jiayu Chi, Qingyun Mo, Mingyue Wang, Yuzhi Sun, Chuanquan Liu, Yanan Zhang, Yue Zhu, Baian Kuang, Chunguang Yan, Haoyang Liu, Ludvig J. Backman, Jialin Chen

**Affiliations:** ^1^ School of Medicine Southeast University Nanjing 210009 P. R. China; ^2^ Center for Stem Cell and Regenerative Medicine Southeast University Nanjing 210009 P. R. China; ^3^ Jiangsu Key Laboratory for Biomaterials and Devices Southeast University Nanjing 210096 P. R. China; ^4^ China Orthopedic Regenerative Medicine Group (CORMed) Hangzhou 310058 P. R. China; ^5^ Department of Orthopaedic Surgery Institute of Digital Medicine Nanjing First Hospital Nanjing Medical University Nanjing 210006 P. R. China; ^6^ Department of Integrative Medical Biology, Anatomy Umeå University Umeå SE‐901 87 Sweden; ^7^ Department of Community Medicine and Rehabilitation, Physiotherapy Umeå University Umeå SE‐901 87 Sweden

**Keywords:** macrophage polarization, proteomics, stiffness, stem cell, tenogenic differentiation

## Abstract

Stiffness is an important physical property of biomaterials that determines stem cell fate. Guiding stem cell differentiation via stiffness modulation has been considered in tissue engineering. However, the mechanism by which material stiffness regulates stem cell differentiation into the tendon lineage remains controversial. Increasing evidence demonstrates that immune cells interact with implanted biomaterials and regulate stem cell behaviors via paracrine signaling; however, the role of this mechanism in tendon differentiation is not clear. In this study, polydimethylsiloxane (PDMS) substrates with different stiffnesses are developed, and the tenogenic differentiation of mesenchymal stem cells (MSCs) exposed to different stiffnesses and macrophage paracrine signals is investigated. The results reveal that lower stiffnesses facilitates tenogenic differentiation of MSCs, while macrophage paracrine signals at these stiffnesses suppress the differentiation. When exposed to these two stimuli, MSCs still exhibit enhanced tendon differentiation, which is further elucidated by global proteomic analysis. Following subcutaneous implantation in rats for 2 weeks, soft biomaterial induces only low inflammation and promotes tendon‐like tissue formation. In conclusion, the study demonstrates that soft, rather than stiff, material has a greater potential to guide tenogenic differentiation of stem cells, which provides comprehensive evidence for optimized bioactive scaffold design in tendon tissue engineering.

## Graphical abstract

Material stiffness influences tenogenic differentiation of stem cells in tendon tissue engineering. Softer materials stimulate tenogenic differentiation of stem cells by inhibiting extracellular signal‐regulated kinases 1/2 signaling pathway, whereas macrophages, in response to these stiffnesses, inhibit tenogenic differentiation via paracrine signaling. Generally, soft, rather than, stiff materials have a greater potential to guide the tenogenic differentiation of stem cells.

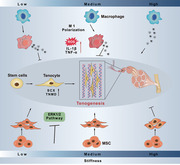

## 1 Introduction

The tendon is a fibrous connective tissue that connects muscle to bone and transmits mechanical forces between these two tissues. Owing to overuse, aging and trauma, the tendon is prone to acute injuries and progressive wear, resulting in the development of tendon diseases. Morphologically, these tendon diseases are accompanied by changes in matrix composition, organization, vascularity, and immune responses.^[^ ^1^ ^]^ Moreover, in the subsequent tendon healing process, disorganized fibrotic scars often fail to retain normal structure and function.^[^ ^2^ ^]^ For decades, high‐quality tendon regeneration has been urgently needed, and recent advances in tendon tissue engineering have brought new hope to those suffering from tendon injuries.

Tendon tissue engineering integrates cells, scaffolds, and bioactive factors to restore or regenerate damaged tendons.^[^ ^3^ ^]^ In tendon tissue engineering, directing stem cells to differentiate into tendon lineages with physicochemical stimuli is significant for tendon repair and regeneration.^[^ ^4^ ^]^ In particular, the physical properties of biomaterials, such as pore size, roughness, topography and stiffness, have gained considerable attention. Material stiffness is a property that has been widely demonstrated to regulate various stem cell behaviors, such as cell adhesion, proliferation, migration, reactive oxygen species (ROS) production and stem cell differentiation.^[^ ^5^ ^]^ Thus, material stiffness is an important parameter in tissue engineering that regulates the differentiation of endogenous and exogenous stem cells. In recent years, several studies have investigated the influence of material stiffness on stem cell differentiation; however, the optimum stiffness for tenogenesis remains controversial. Liu et al. discovered that soft gelatin hydrogels with a stiffness of 2.34 ± 1.48 kPa robustly promoted tenogenesis.^[^ ^5c^ ^]^ In addition, Sharma et al. reported that moderate stiffness (30–50 kPa) more effectively supported the tenogenic behaviors of bone marrow stromal cells compared with softer or higher stiffness (<100 kPa).^[^ ^5^, ^6^ ^]^ However, several other studies have reported that stiffer substrates (≈100 kPa to ≈8 GPa) stimulate stem cell differentiation toward the tendon lineage.^[^ ^5^, ^7^ ^]^ Further studies are warranted to determine the optimal stiffness of biomaterials to guide the tenogenic differentiation of stem cells.

In addition, tendon injuries and artificial transplants often trigger host inflammatory responses, resulting in various tendon pathologies (e.g., fibrotic scar formation and tendon adhesion) that impair functional tendon recovery.^[^ ^8^ ^]^ Macrophages are one of the earliest immune cells to arrive at injured sites and interact with transplanted biomaterials to drive inflammation.^[^ ^9^ ^]^ Moreover, the sensitivity of macrophages to biomaterials is more profound than that of tenocytes, which are the main cell population in tendons.^[^ ^10^ ^]^ To date, material stiffness has been proven to affect various macrophage behaviors, including migration, oxidative stress, polarization and functional expression.^[^ ^11^ ^]^ Stiffness‐activated macrophages can interact with the surrounding cells via paracrine signaling, which can potentially influence the tenogenic differentiation of endogenous or exogenous stem cells during tendon repair and regeneration in vivo.^[^ ^12^ ^]^ Therefore, when a scaffold with appropriate stiffness is used to guide tenogenesis in tendon tissue engineering, the paracrine signals of macrophages in response to stiffness should be considered. Nevertheless, the mechanism by which macrophages respond to material stiffness and affect stem cells has not yet been determined. Critically, paracrine signaling can act synergistically or antagonistically on tenogenesis, thereby influencing tendon repair and regeneration in stem cell‐based tendon tissue engineering. Therefore, it is important to comprehensively investigate how material stiffness influences the functional status of macrophages and how these macrophages regulate tenogenic differentiation of stem cells via paracrine signaling.

In this study, we fabricated polydimethylsiloxane (PDMS) substrates with different stiffnesses by controlling the ratio of the base and curing agents. Mesenchymal stem cells (MSCs) were seeded on these substrates to examine the effect of material stiffness on tenogenic differentiation. Subsequently, we exposed RAW 264.7 macrophages to substrates with different stiffnesses and investigated their oxidative stress, polarization status and functional expression, as well as how stiffness‐induced macrophage paracrine signals modulate MSCs tenogenesis. Furthermore, we evaluated the combined effects of material stiffness and macrophage paracrine signals on the tenogenic differentiation of MSCs and investigated the underlying mechanisms using global proteomic analysis. Finally, heterotopic transplantation was performed to validate the potential role of material stiffness in tendon‐like tissue formation in vivo.

## 2 Results and Discussion

### 2.1 Material Stiffness Regulates Cell Behaviors of MSCs In Vitro

**Figure 1:**
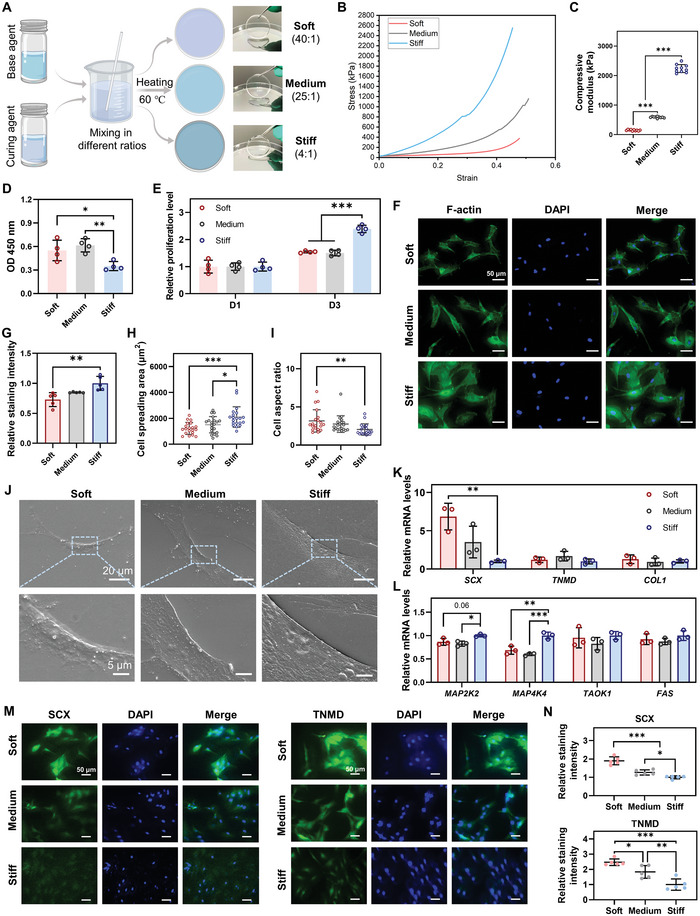
Material stiffness regulates cell behaviors of MSCs in vitro. A) Schematic illustration of the preparation of PDMS with different stiffnesses. B) Typical strain–stress curves of the three PDMS substrates with different stiffnesses. C) Compressive modulus of the three PDMS substrates with different stiffnesses (*n* = 10). D) Cell adhesion of MSCs 24 h after seeding, evaluated by measuring optical density (OD) 450 nm (*n* = 4). E) Cell proliferation of MSCs evaluated by cell counting kit‐8 (CCK‐8) assay (*n* = 4). F,G) Representative images and relative intensity of F‐actin staining in MSCs on day 1 (*n* = 5). Scale bars = 50 µm. H,I) Cell spreading area and cell aspect ratio of MSCs calculated from F‐actin staining images (*n* = 20). J) SEM images of the three PDMS substrates seeded with MSCs. Low magnification: scale bars = 20 µm; High magnification: scale bars = 5 µm. K) Gene expression of tenogenic makers in MSCs on day 3 (*n* = 3). L) Gene expression of mitogen‐activated protein kinases (MAPK) pathway‐related genes in MSCs on day 3 (*n* = 3). M,N) Representative images and quantitative analysis of IF staining for SCX and TNMD on day 5 (*n* = 5). Scale bars = 50 µm. The results are shown as the mean ± SD. *p < 0.05; **p < 0.01; ***p < 0.001 determined using one‐way ANOVA with Tukey's post hoc test (C–E, G–I, K, L, and N).

As shown in **Figure** **1**A, base and curing agents with ratios of 40:1, 25:1 and 4:1 were used to prepare soft, medium and stiff PDMS substrates, respectively. Mechanical compression tests were performed to evaluate their mechanical properties, which confirmed that PDMS substrates with different stiffnesses were successfully fabricated (Figure 1B). PDMS in the Soft, Medium and Stiff groups had a compressive modulus of 149.53 ± 21.83 kPa, 589.99 ± 24.53 kPa and 2.36 ± 0.14 MPa, respectively, which covered a physiological stiffness ranging from muscle to tendon (Figure 1C, and Figure S1, Supporting Information). Similar to previous material stiffness researches, the moduli of these substrates were close to a geometric sequence with a common ratio of 4, which allowed for a wide stiffness range and sufficient difference among groups.^[^ ^5^, ^13^ ^]^ To increase hydrophilicity and facilitate cell adhesion, all PDMS substrates were coated with 0.1 mg mL^−1^ Collagen I (COL1). The surface morphologies of the PDMS substrates before and after COL1 coating were evaluated using scanning electron microscopy (SEM). The SEM images showed that all PDMS substrates had smooth surfaces before COL1 coating, whereas collagen filaments were extensively distributed over the PDMS surfaces in all groups after COL1 coating, leading to the rough but similar surfaces of all PDMS substrates (Figure S2, Supporting Information). A water contact angle test was performed to evaluate the hydrophilicity of the PDMS substrates. Our results revealed that the COL1 coating substantially improved the hydrophilic properties of all PDMS substrates, and the COL1‐coated PDMS in the Soft, Medium and Stiff groups had water contact angles of 51.64°, 27.48° and 38.10°, respectively, favorable for cell culture (Figure S3, Supporting Information).^[^ ^14^ ^]^ Brown et al. revealed that seeded cells could change the mechanical properties of biomaterials, such as collagen gel.^[^ ^15^ ^]^ Thus, we performed mechanical compression tests on PDMS substrates with and without COL1 coating and MSCs seeding. The results revealed that the three PDMS substrates before and after COL1 coating and MSCs seeding had similar compressive moduli, indicating that both the COL1 coating and MSCs seeding did not significantly influence the mechanical properties of the three PDMS substrates (Figure S4, Supporting Information). Taken together, our results confirmed that PDMS substrates with different stiffnesses but similar surface properties were successfully fabricated.

Subsequently, we evaluated the influence of different stiffnesses on the MSCs’ behavior. First, a CCK‐8 assay was conducted to evaluate the adhesion and proliferation of MSCs on different substrates (Figure 1D,E). After MSCs adhered to different substrates for 24 h, a significantly higher value of OD 450 was observed in the Soft and Medium groups than that in the Stiff group. The result indicated that the softer substrates promoted cell adherence at the early stage of cell–material interaction (Figure 1D), which could be attributed to the combined effects of their different stiffnesses and hydrophilicity (Figure 1C, and Figure S3, Supporting Information). After normalization of the different cell adhesions between groups, we found that the ratio of MSCs proliferation was significantly higher on the stiff substrate than that on softer substrates on day 3 (Figure 1E), which was consistent with previous studies.^[^ ^16^ ^]^ Subsequently, F‐actin staining was performed to investigate the effect of various stiffnesses on the spreading and morphology of MSCs (Figure 1F–I). After 24 h, MSCs in the Soft and Medium groups exhibited a long spindle shape with a lower fluorescence intensity, whereas MSCs in the Stiff group showed a spreading morphology with a higher fluorescence intensity (Figure 1F,G). Increased actin filament formation (as indicated by more intense fluorescence) induced by higher stiffness has been reported to facilitate various cell behaviors such as cell spreading and migration.^[^ ^17^ ^]^ Quantitative analysis revealed that MSCs cultured on softer substrates exhibited significantly decreased cell spreading area but increased cell aspect ratio compared to the Stiff group (Figure 1H,I). The average cell spreading area of the Stiff group was 1.72 times that of the Soft group and 1.39 times that of the Medium group; the average area of the Medium group exceeded that of the Soft group by 1.24 times (Figure 1H). We further evaluated how MSCs interacted with PDMS substrates with different stiffnesses using SEM. The results revealed that MSCs adhered well to the surface of the three substrates and displayed increased cell spreading area on the substrates with higher stiffness (Figure 1J), which was consistent with the results of F‐actin staining (Figure 1F). Collectively, these results revealed that stiffness could influence the spreading and morphology of MSCs by regulating actin formation, as previously described.^[^ ^5c^ ^]^ Moreover, previous studies have demonstrated that cell morphology directed by material stiffness suggests lineage specification of stem cells, which is initiated by focal adhesion and cytoskeletal organization changes by activating RhoA/ROCK/ERK1/2 signaling pathways.^[^ ^18^ ^]^ MSCs cultured on the soft substrate exhibited a more similar shape to physiological tenocytes as compared to medium and stiff substrates, possibly suggesting a higher potential of soft substrates to direct the tenogenic differentiation of MSCs.

To further evaluate the effect of substrate stiffness on tenogenic differentiation, we evaluated the expression profile of MSCs following culture under different stiffness conditions. After 3 d, MSCs in the Soft group had significantly higher gene expression of *SCX*, a marker of early tenogenic differentiation, than those in the Stiff group, indicating that lower stiffness induced tenogenic differentiation of MSCs (Figure 1K). Immunofluorescence (IF) staining for SCX and TNMD (a marker of terminal tenogenic differentiation) confirmed that softer substrates significantly enhanced the protein expression of SCX and TNMD, thereby inducing MSCs differentiation toward the tendon lineage (Figure 1M,N). In addition, several genes associated with MAPK pathways, such as *MAP2K2* and *MAPK4K4*, showed significantly decreased expression in the Soft and Medium groups compared to the Stiff group (Figure 1L). MAP2K2, also known as MEK2, is an upstream gene of the extracellular signal‐regulated kinase (ERK) MAPK pathway, a vital pathway for osteogenic differentiation of MSCs on stiff substrates.^[^ ^19^ ^]^ MAPK4k4 is involved in various cell functions and biological or pathological processes, and it has been reported to regulate multiple MAPK cascades, including ERK1/2.^[^ ^20^ ^]^ Thus, the enhanced tenogenic differentiation of MSCs on softer substrates could be partially explained by the inhibition of ERK1/2 signaling pathway. Consistent with our results, Liu et al. found that lower stiffness promoted the expression of tendon‐related markers (*SCX*, *TNMD*, and *THBS4*) by inhibiting focal adhesion kinase (FAK) and ERK1/2 signaling pathways.^[^ ^5c^ ^]^ A recent study by Hou et al. also demonstrated that inhibition of the ERK1/2 signaling pathway resulted in enhanced tenogenic differentiation of tendon‐derived stem cells (TDSCs).^[^ ^21^ ^]^ Taken together, our results indicate that lower stiffness promotes tenogenic differentiation of MSCs, which could be attributed to changes in morphology and inhibition of the ERK1/2 signaling pathway.

In the past, several studies have concluded that stiffer materials with a stiffness ranging from kilopascals to gigapascals promoted stem cells to differentiate into the tendon lineage as compared to softer materials.^[^ ^5^, ^7^ ^]^ In contrast, other studies have demonstrated that soft material with a stiffness of less than 100 kPa supported tenogenic differentiation of stem cells and that moderate stiffness (30–50 kPa) could mimic the physiological stiffness of the tendon and effectively induce stem cell differentiation toward the tendon lineage.^[^ ^5^, ^6^ ^]^ Similarly, our results support that softer, rather than stiff, materials direct stem cell differentiation into the tendon lineage.

### 2.2 Macrophage Polarization in Response to Material Stiffness Influences Tenogenic Differentiation of MSCs via Paracrine Signaling

It is well known that the inflammatory response in the healing process determines whether tendon repair and regeneration are successful following the in vivo implantation of biomaterials.^[^ ^22^ ^]^ Macrophages are among the fastest immune cells to respond to implanted biomaterials.^[^ ^10^ ^]^ Moreover, macrophages sense the physical properties of biomaterials (e.g., fiber arrangement) and can initiate macrophage polarization toward the M1 or M2 phenotype to induce a pro‐inflammatory or anti‐inflammatory environment, respectively.^[^ ^22^, ^23^ ^]^ In particular, material stiffness has been found to regulate migration, oxidative stress, polarization and functions of macrophages, as well as the expression and secretion of inflammatory factors.^[^ ^11^, ^12^ ^]^ The secreted factors from polarized macrophages have been shown to, through paracrine signaling, regulate behaviors of surrounding cells, such as immunomodulation of MSCs and tendon‐related gene expression in tenocytes.^[^ ^10^, ^12^, ^22^ ^]^ Thus, in addition to the effects of material stiffness itself on stem cells, macrophage paracrine signals in response to material stiffness also play a crucial role in determining stem cell fate in tendon tissue engineering. This aspect should be further investigated to obtain an objective and comprehensive effect of the material stiffness on stem cell differentiation.

#### 2.2.1 Material Stiffness Modifies the Oxidative Stress, Polarization Status, and Inflammatory Factor Secretions of Macrophages

**Figure 2:**
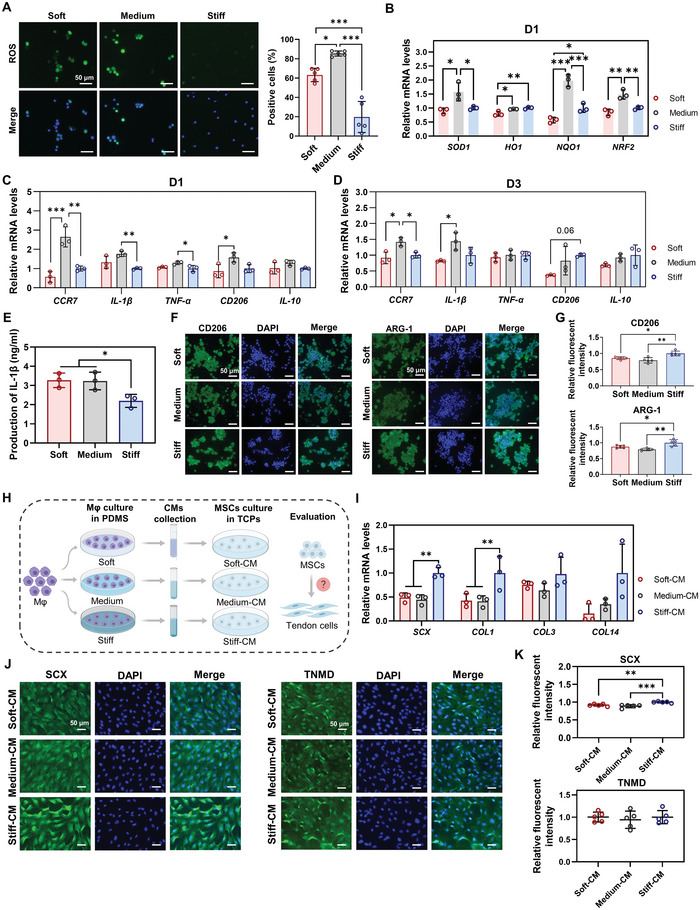
Macrophage polarization in response to material stiffness influences the tenogenic differentiation of MSCs by paracrine signals. A) Representative images and quantitative analysis of ROS staining in macrophages on day 1 (*n* = 5). B) Gene expression of antioxidant mediators in macrophages on day 1 by qPCR (*n* = 3). C,D) Gene expression of pro‐inflammatory genes (M1 markers: *CCR7*, *IL‐1β*, and *TNF‐α*) and anti‐inflammatory genes (M2 markers: *CD206* and *IL‐10*) in macrophages by qPCR on days 1 and 3 (*n* = 3). E) Production of IL‐1*β* in macrophages on day 1 (*n* = 3). F,G) Representative images and quantitative analysis of IF staining for CD206 and ARG‐1 in macrophages on day 3 (*n* = 5). H) Schematic illustration of the processes to investigate macrophage paracrine signals on tenogenic differentiation of MSCs. *Mφ*: macrophages. I) Gene expression of tenogenic markers in MSCs treated with different conditional media (CMs) by qPCR (*n* = 3); CMs were collected from the supernatant of the culture medium of macrophages on different PDMS substrates on day 1. J,K) Representative images and quantitative analysis of IF staining for SCX and TNMD in MSCs on day 5 (*n* = 5). Scale bars = 50 µm. The results are shown as the mean ± SD. *p < 0.05; **p < 0.01; ***p < 0.001 determined using one‐way ANOVA with Tukey's post hoc test (A–E, G, I, and K).

ROS signaling, which is involved in the regulation of the functional status and phenotype of macrophages, has been found to vary depending on the material stiffness.^[^ ^11^, ^24^ ^]^ Increased ROS production and the accompanying oxidative stress in macrophages can serve as a secondary inflammatory response that triggers the downstream inflammatory signals and thereby influence the polarization status and the secretion profile of macrophages.^[^ ^11d^ ^]^ Thus, we first evaluated the influence of stiffness on ROS production and oxidative stress in macrophages. ROS staining was conducted to determine the ROS levels in macrophages exposed to the three stiffness substrates for 1 d. Fluorescence images and quantitative analysis revealed that macrophages in the Soft and Medium groups had significantly more ROS‐positive cells than those in the Stiff group, indicating that more ROS accumulated in the macrophages cultured on softer substrates (**Figure** **2**A). Compared to the soft substrate, macrophages on the medium substrate exhibited notably increased ROS production (Figure 2A). The expression of antioxidant mediators, including *SOD1*, *HO1*, *NQO1* and *NRF2* was evaluated using quantitative real‐time PCR (qPCR). Notably, we observed that MSCs in the Medium group had significantly higher expression of *SOD1*, *NQO1*, and *NRF2* compared to the other two groups on day 1 (Figure 2B). However, the differences in gene expression disappeared on day 3, suggesting that the increased oxidative stress in macrophages only occurred in the early stage of macrophage–material interaction (Figure S5, Supporting Information). Collectively, these results demonstrate that softer materials (especially the one with medium stiffness) trigger increased ROS accumulation and subsequently increase oxidative stress in macrophages. Consistent with our results, previous studies have also reported that softer substrates triggered higher cytoplasmic and mitochondrial ROS in macrophages, which could serve as a secondary inflammation signal to induce macrophage polarization toward the M1 phenotype.^[^ ^11b,d^ ^]^

We further investigated whether the polarization status of macrophages was affected by different stiffnesses. The gene expression profiles of pro‐inflammatory genes (M1 markers: *CCR7*, *IL‐1β*, and *TNF‐α*) and anti‐inflammatory genes (M2 markers: *CD206* and *IL‐10*) were examined in macrophages cultured on different stiffnesses for 1 and 3 d (Figure 2C,D). Notably, macrophages in the Medium group had significantly increased expression of M1 markers, including *CCR7*, *IL‐1β* and *TNF‐α*, compared to the Soft and/or Stiff groups on both days 1 and 3 (Figure 2C,D). ELISA for IL‐1*β* revealed that more IL‐1*β* was produced and secreted by macrophages exposed to soft and medium stiffnesses than those exposed to stiff stiffness (Figure 2E). These results indicate higher pro‐inflammatory properties of macrophages cultured on softer substrates. In addition, although higher M2 marker (*CD206* and *IL‐10*) expression was also observed in macrophages in the Medium group on day 1, the expression of these genes seemed to decrease compared to the Stiff group on day 3 (Figure 2C,D). IF for CD206 and ARG‐1 was performed to clarify the M2 marker expression of macrophages in response to different stiffnesses. We discovered that the macrophages cultured on softer substrates had a significantly lower fluorescence intensity of CD206 and ARG‐1 than those on the stiff substrate, confirming their decreased M2 marker expression (Figure 2F,G). Together, these results indicated that softer substrates were inclined to stimulate macrophages to switch to the M1 phenotype, which was consistent with the changes in ROS production (Figure 2A). Specifically, despite the similar IL‐1*β* production in the Soft and Medium groups, the pro‐inflammatory property of macrophages on the soft substrate was believed to be weaker than that on the Medium substrate, given its lower ROS production, oxidative stress levels and M1 marker expression (Figure 2A–D). Collectively, our findings demonstrated that softer substrates (especially medium substrate) promoted ROS production and oxidative stress activation in macrophages, thereby inducing macrophage polarization toward the M1 phenotype. Consistently, Chen et al. demonstrated that low material stiffness (≈2.55 kPa) promoted macrophage polarization into the M1 phenotype through activation of the NF‐*κ*B signaling pathway as compared to medium (≈34.88 kPa) and stiff materials (≈63.53 kPa).^[^ ^11b^ ^]^ Similarly, Friedemann and colleagues found that macrophages cultured on stiffer material (≈57.5 to ≈118.5 kPa) showed an anti‐inflammatory phenotype compared to macrophages cultured on soft material (≈27.1 kPa).^[^ ^11c^ ^]^ In contrast, previous studies have also reported that soft materials promoted macrophage polarization toward the M2 phenotype, whereas stiff materials induced a switch of macrophages to the M1 phenotype.^[^ ^11^, ^25^ ^]^ These controversies on stiffness‐mediated macrophage polarization could be caused by different cell sources, stiffness range and measurements, culture dimensions, and other factors. Actually, the soft PDMS with the stiffness of ≈149.53 kPa in our study could, in relation to other studies, be considered as a “medium” or “stiff” substrate which induced M1 phenotype polarization of macrophages.^[^ ^11^, ^12^, ^25^ ^]^ In addition, the macrophages in the Medium group also exhibited notably higher pro‐inflammatory expression compared to the Soft group (Figure 2C,D). Our findings suggest that the response of macrophages to material stiffness is a non‐monotonic behavior and that “moderate” stiffness could be sensed by macrophages to trigger inflammation and induce polarization into the M1 phenotype.

#### 2.2.2 Paracrine Signals by Macrophages in Response to Material Stiffness Regulate Tenogenic Differentiation of MSCs

Our results suggest that softer material induces macrophage polarization toward the M1 phenotype, which secretes more pro‐inflammatory factors, thereby forming or enhancing an inflammatory environment in the injured tendon. The increased inflammatory environment could influence the behavior of the surrounding cells, such as tendon stem cells and tenocytes.^[^ ^10^ ^]^ Higher levels of pro‐inflammatory factors (e.g., IL‐1*β* and TNF‐*α*) have been reported to irreversibly suppress tenogenic differentiation of stem cells.^[^ ^26^ ^]^ Thus, we hypothesized that the paracrine signals of M1 macrophages on softer materials could inhibit the tenogenic differentiation of MSCs.

To test the above hypothesis, CMs from macrophages cultured on different PDMS substrates for 1 d were collected (Figure 2H). qPCR and IF staining were performed to evaluate tenogenic differentiation of MSCs after exposure to different CMs. After culturing MSCs with CMs derived from macrophages for 3 d, we observed that the gene expression of tendon‐related markers, including *SCX* and *COL1* in the Soft‐CM and Medium‐CM groups was significantly lower than that in the Stiff‐CM group (Figure 2I). *COL3* and *COL14* showed similar trends, although no significant difference was detected between the groups (Figure 2I). After culturing MSCs with different CMs for 5 d, IF staining for SCX and TNMD was conducted to further evaluate tenogenic differentiation. Our results showed that the Soft‐CM and Medium‐CM groups exhibited less intense staining for SCX than did the Stiff‐CM group (Figure 2J). Quantitative analysis further confirmed that CMs derived from macrophages in the Soft‐CM and Medium‐CM groups suppressed the tenogenic differentiation of MSCs compared to the Stiff‐CM group (Figure 2K). Collectively, these findings demonstrated that the paracrine signals of M1 macrophages cultured on softer substrates significantly inhibited tenogenic differentiation of MSCs (Figure 2I–K). Interestingly, the opposite effect was observed when MSCs were simply exposed to softer substrates, that is, softer substrates stimulated the tenogenic differentiation of MSCs (Figure 1K–N).

Similar to our results, previous studies have found that macrophages respond to other physical properties of materials, such as topography and mechanical loading, which affect the polarization status of macrophages and subsequently influence tendon‐related gene expression in tenocytes.^[^ ^10^, ^22^ ^]^ Schoenenberger and colleagues found that the paracrine signals of M1 macrophages in response to matrix topography inhibited the expression of tendon markers in tenocytes in vitro.^[^ ^22a^ ^]^ Another study by Schoenenberger et al. discovered that M1 polarization of macrophages, induced by dynamic mechanical loading, resulted in decreased expression of tendon‐related genes in tenocytes, such as *SCX*, *COL1* and *COL3* via paracrine signaling.^[^ ^10^ ^]^ In addition, several other studies have revealed that material stiffness regulates the paracrine signals of macrophages and MSCs, which influences the differentiation and immunomodulation potential of MSCs.^[^ ^12^ ^]^ Taken together, macrophages sense the stiffness of materials, and softer materials induce macrophage polarization toward the pro‐inflammatory phenotype that inhibits MSCs’ tenogenic differentiation by paracrine signaling.

### 2.3 Influence of Simultaneous Stimulation of Material Stiffness and Macrophage Paracrine Signals on Tenogenic Differentiation of MSCs and its Underlying Mechanisms

#### 2.3.1 Tenogenic Differentiation of MSCs when Simultaneously Stimulated by Stiffness and Macrophage Paracrine Signals

As mentioned above, material stiffness and macrophage paracrine signals have diverse effects on tenogenic differentiation of MSCs. Softer materials stimulated MSCs’ tenogenic differentiation, whereas CMs from macrophages cultured on softer materials inhibited MSCs’ tenogenic differentiation. Thus, in vivo, with implanted soft materials containing MSCs, the presence of paracrine signals from macrophages could induce an antagonistic effect on tenogenic differentiation. Such antagonistic signals by macrophages could disturb or even reverse the positive role of the designed scaffolds in stem cell differentiation and thus impair tendon regeneration. Therefore, it is crucial to evaluate the tenogenic differentiation of MSCs on materials with different stiffnesses in the presence of paracrine signals from macrophages.

**Figure 3:**
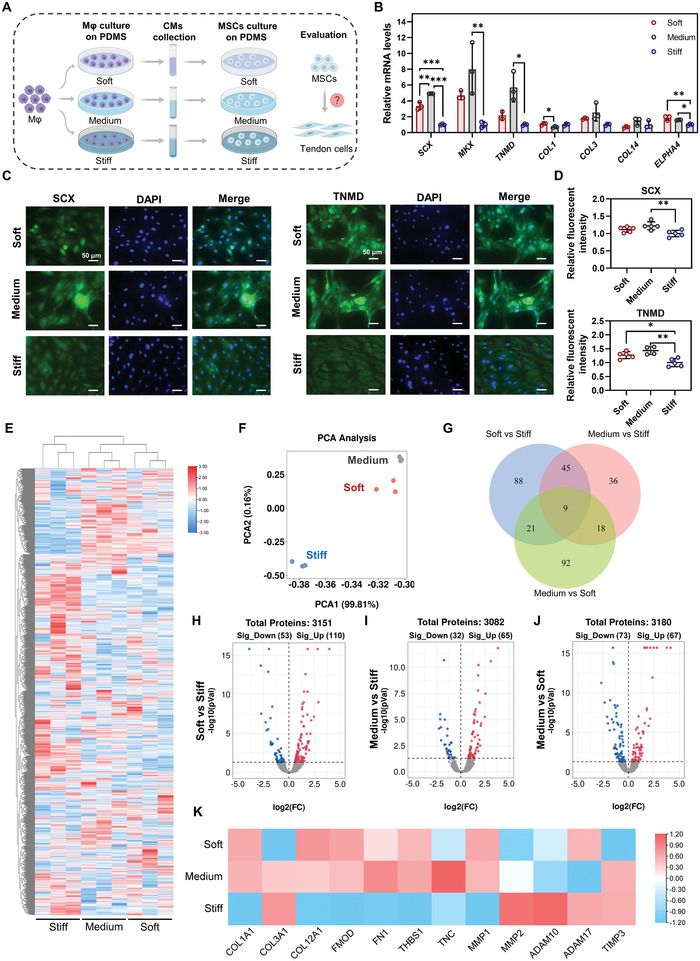
Influence of simultaneous stimulation by material stiffness and macrophage paracrine signals on tenogenic differentiation of MSCs. A) Schematic illustration of the processes to investigate the influence of simultaneous stimulation by stiffness and macrophage paracrine signals on tenogenic differentiation of MSCs. *Mφ*: macrophages. B) Gene expression of tenogenic makers in MSCs on day 3 (*n* = 3). C,D) Representative images and quantitative analysis of IF staining for SCX and TNMD in MSCs on day 5 (*n* = 5). Scale bars = 50 µm. E) Heatmap of all identified protein expressions in three groups after cluster analysis. F) Principal component analysis (PCA) plot of proteomic data in three groups. G) Venn diagram of all differentially expressed proteins (DEPs) in paired comparison. DEPs were defined as proteins with a *p*‐value less than 0.05. H–J). Volcano plot of all protein expressions in Soft versus Stiff, Medium versus Stiff, and Medium versus Soft. K) Protein expression of tendon‐related proteins from proteomic profiles. The results are shown as the mean ± SD. *p < 0.05; **p < 0.01; ***p < 0.001 determined using one‐way ANOVA with Tukey's post hoc test (B,D).

We collected CMs from macrophages cultured at different stiffnesses for 1 d, and the collected CMs were later used to culture MSCs seeded at different stiffnesses (**Figure** **3**A). The specific stiffness used to collect the macrophage CM was matched with the stiffness used to culture MSCs; that is, CM from soft PDMS was used when culturing MSCs on soft PDMS (Figure 3A). After 3 d of culture, MSCs in the Soft and Medium groups had significantly higher gene expression of tenogenic markers than those in the Stiff group, including *SCX*, *MKX*, *TNMD* and *ELPHA4* (Figure 3B). Moreover, we observed that the Medium group showed up‐regulated expression of these genes compared to the Soft group; however, only SCX was significantly up‐regulated. This trend indicated that the Medium group induced slightly enhanced tenogenic differentiation of MSCs compared to the Soft group (Figure 3B). IF staining for SCX and TNMD was conducted to further clarify the differentiation status of MSCs on day 5. Fluorescence images and quantitative analysis revealed that MSCs in the Soft and Medium groups with macrophage CMs from matched stiffnesses exhibited increased tenogenic differentiation (Figure 3C,D). No significant difference was observed between the Soft and Medium groups (Figure 3C,D). Collectively, although CMs from macrophages cultured on softer substrates had a negative effect on tenogenic differentiation, the MSCs on these substrates still exhibited enhanced tenogenic differentiation compared to the stiff one. These results indicate that the strong capacity of lower stiffness to stimulate tenogenic differentiation of stem cells covered or reversed the negative influence of macrophage paracrine signals. Interestingly, considering the superior performance of the soft substrate over the medium substrate in enhancing tenogenic differentiation and the similar level of inhibited tenogenic differentiation by the matched CMs, it was reasoned that higher tenogenic differentiation of MSCs should be observed in the Soft group. However, inconsistent results were observed for the expression of the analyzed tenogenic genes and proteins. Thus, we speculate that more complicated interactions occur, which should be further investigated to better understand the functional status of MSCs.

#### 2.3.2 Proteomic Analysis to Understand the Functional Status of MSCs when Simultaneously Stimulated by Material Stiffness and Macrophage Paracrine Signals

To further understand the functional status of MSCs treated with different stiffnesses and CMs simultaneously, proteomic analysis was performed to obtain a global view of the affected biological processes and signaling pathways within MSCs (Figure 3A). After 3 d of culture, global cellular protein expression of MSCs was examined using label‐free quantification (LFQ)‐based proteomic analysis.

As shown in Figure 3E, the heatmap of all identified proteins after cluster analysis showed a notable difference between the three groups. A similar protein expression profile was observed between the Medium and Soft groups. PCA showed that the replicates of each group were assembled into a clear group, and similar features were found in the Soft and Medium groups (Figure 3F). These results indicated that similar cellular responses of MSCs were initiated in the Soft and Medium groups compared to the Stiff group. We further investigated the changes in protein expression in the different groups by paired comparison, and the changed protein with a *p*‐value < 0.05 was recognized as a differentially expressed protein (DEP). In Soft versus Stiff, 163 DEPs were identified, with 110 proteins upregulated and 53 downregulated (Figure 3H). When comparing the Medium and Stiff groups, we recognized that 97 proteins were differentially expressed, including 65 upregulated and 32 downregulated DEPs (Figure 3I). A total of 140 DEPs were identified in Soft versus Medium, with 67 proteins upregulated and 73 downregulated (Figure 3J). The Venn diagram of DEPs revealed that only nine DEPs were shared in all paired comparisons, and 88, 36 and 92 DEPs were uniquely shown in Soft versus Stiff, Medium versus Stiff and Medium versus Soft, respectively (Figure 3G). Subsequently, the expression of tendon‐related proteins was investigated in the proteomic profiles of MSCs, and we observed that several tendon‐lineage proteins in the Soft and Medium groups had notably higher expression compared to the Stiff group, including COL1A1, COL12A1, FMOD, FN1 and THBS1 (Figure 3K), confirming the enhanced tenogenic differentiation of MSCs in these two groups (Figure 3B–D). In addition, several matrix degradation enzymes were differentially expressed and were possibly involved in the matrix remodeling of differentiated MSCs into the tendon lineage (Figure 3K). Collectively, these results support the increased tenogenic differentiation of MSCs when exposed to softer stiffness and corresponding CMs (Figure 3B–D,K). Furthermore, notable changes were observed in these three groups, suggesting that different mechanisms regulate stem cell differentiation. Thus, functional analysis of the DEPs in paired comparisons was performed to elucidate the related cellular processes and signaling pathways.

**Figure 4:**
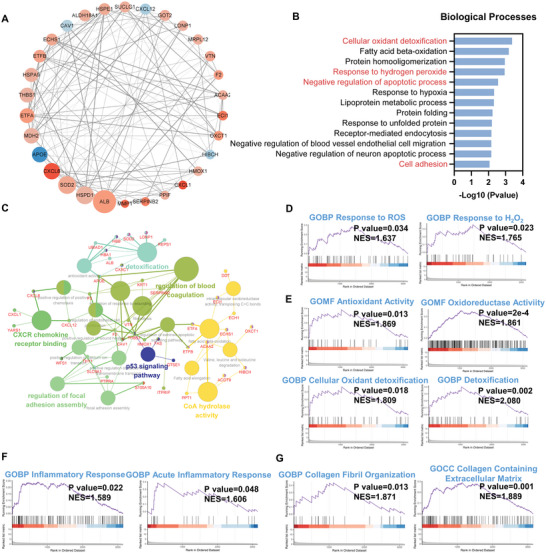
Functional changes of MSCs in Soft versus Stiff group under simultaneous stimulation of material stiffness and macrophage paracrine signals. A) Protein interaction network for the 30 hub DEPs in Soft versus Stiff. The larger size of the protein node indicated a higher frequency in the protein network. The up‐regulated proteins are presented in red while the down‐regulated are in blue. A deeper color of nodes represents a lower or higher fold change of DEPs. B) Key GO terms significantly enriched in BP (Soft versus Stiff). C) Network for GO and KEGG terms enriched from all DEPs (Soft versus Stiff). D) GSEA plot of cellular response to ROS and H_2_O_2_ (Soft versus Stiff). E) GSEA plot of GO terms associated with antioxidant responses (Soft versus Stiff). F) GSEA plot of GO terms associated with inflammatory responses (Soft versus Stiff). G) GSEA plot of GO terms associated with collagen formation and organization (Soft versus Stiff).

*Functional changes of MSCs in Soft versus Stiff group with simultaneous stimulation of material stiffness and macrophage paracrine signals*: The protein interaction network for all DEPs when comparing the Soft and Stiff groups is shown in Figure S6A, Supporting Information. Next, the 30 hub DEPs that could potentially play a critical role in the altered cellular processes and pathways were screened using CytoHubba in Cytoscape (**Figure** **4**A). Among these proteins, 26 of the 30 hub DEPs were upregulated, and only 4 of them were downregulated (Soft versus Stiff), suggesting that cellular processes and pathways of MSCs in the soft group were more likely to be activated. Notably, we observed that several oxidative stress‐related proteins were differentially expressed, with upregulated SOD2, HMOX1 (HO‐1), ALB and LONP1, as well as downregulated APOE (Soft versus Stiff) (Figure 4A). In addition, a few proteins associated with inflammation were also changed, with upregulated CXCL1 and CXCL8, as well as downregulated CXCL12 (Soft versus Stiff) (Figure 4A). These results suggested that oxidative stress and inflammation could be initiated in the MSCs in the Soft group compared to the Stiff group. Gene ontology (GO) enrichment analysis for all DEPs was conducted to further determine the affected biological processes (BP), cellular components (CC), and molecular function (MF) in MSCs (Figure 4B, and Figure S6B,C, Supporting Information). The enriched GO terms were narrowed according to the higher significance (*p* < 0.05). The top GO terms for BP are shown in Figure 4B. Several associated biological processes were enriched, including cellular oxidant detoxification, response to hydrogen peroxide (H_2_O_2_), negative regulation of the apoptotic process and cell adhesion. Consistent with the changes in BP, we found that antioxidant activity was enriched in MF, whereas only a few associated terms in CC were identified, including extracellular exosome, extracellular matrix and extracellular space (Figure S6B,C, Supporting Information). The network formed by ClueGO in Cytoscape showed the significant terms enriched in BP, CC, MF and KEGG (Kyoto Encyclopedia of Genes and Genomes) pathways, as well as the proteins responsible for the enriched terms (Figure 4C). We observed that several GO terms were significantly enriched, including detoxification, CXCR chemokine receptor binding, regulation of focal adhesion assembly, regulation of blood coagulation, P53 signaling and CoA hydrolase (Figure 4C). Notably, oxidative stress and antioxidant activities seemed to be the primary cellular responses of MSCs exposed to a soft substrate and the corresponding CM. Subsequently, gene set enrichment analysis (GSEA) was performed to clarify cellular changes. The results confirmed that the response to ROS and H_2_O_2_ was significantly enhanced when comparing the Soft and Stiff groups, which followed the activation of antioxidant responses, including antioxidant activity, oxidoreductase activity, cellular oxidant detoxification and detoxification (Figure 4D,E). In addition to oxidative stress, an activated inflammatory response of MSCs was also observed in the soft group; however, no typical pro‐inflammatory biological processes or signaling pathways were identified (Figure 4F). Thus, we reasoned that the activated inflammation in MSCs cultured on soft substrates could be mediated by the enhanced oxidative stress induced by CM from M1 macrophages. ROS production and antioxidant activity of MSCs cultured with different CMs were evaluated using ROS staining and qPCR, respectively. ROS staining and quantitative analysis revealed that the CM in the Soft group significantly promoted ROS production in MSCs compared to the Stiff group on day 1 (Figure S7A, Supporting Information). It was observed that MSCs in the Soft group exhibited increased gene expression of antioxidant mediators, such as *SOD2* and *NRF2*, compared to the Stiff group on day 1, but the differences were not significant (Figure S7B, Supporting Information). From the proteomic profiles, the expression of several antioxidant mediators, including SOD2, HMOX1, LONP1, HBB, HBA1 and ALB, were significantly increased in the Soft group compared to that in the Stiff group (Figure S6D, Supporting Information). It could be speculated that the pro‐inflammatory secretions by macrophages in the Soft group subsequently resulted in excessive production of ROS, which was released into CM and that CM with a high concentration of ROS could potentially activate oxidative stress in MSCs (Figure 2A,B,E). Collectively, these results suggested that CM in the Soft group promoted ROS production and oxidative stress activation in MSCs, which subsequently activated the inflammatory response. It is worth noting that the inflammation in MSCs in the Soft group seemed to be limited, only with an increased expression of *IL‐1β* on day 1 identified by qPCR (Figure S8, Supporting Information).

In addition, several stiffness‐regulated biological processes were also observed when comparing the Soft and Stiff groups, such as cell adhesion and regulation of focal adhesion assembly (Figure 4B,C). Besides, Rho protein signal transduction, which involves the FAK/ROCK/ERK1/2 signaling pathway, was downregulated in the soft group compared to the stiff group (Figure S6E, Supporting Information). Focal adhesion is a complex that can anchor cells to extracellular matrix and function as sensors to transmit mechanical forces and signals from extracellular stiffness.^[^ ^27^ ^]^ Rho protein is known to regulate focal adhesion formation and the actin filament bundles, which are closely associated with this binding process.^[^ ^28^ ^]^ All these changes in biological processes indicated that MSCs sensed and responded to substrate stiffness. Our results demonstrated that under the simultaneous stimulation of substrate stiffness and macrophage paracrine signals, the response of MSCs to stiffness still played a vital role in cellular activity. Consistent with our results in Figure 1L, these results confirmed that soft substrates could potentially promote tenogenic differentiation of MSCs when cultured with CMs by inhibiting stiffness‐sensitive ERK1/2 signaling pathways.

**Figure 5:**
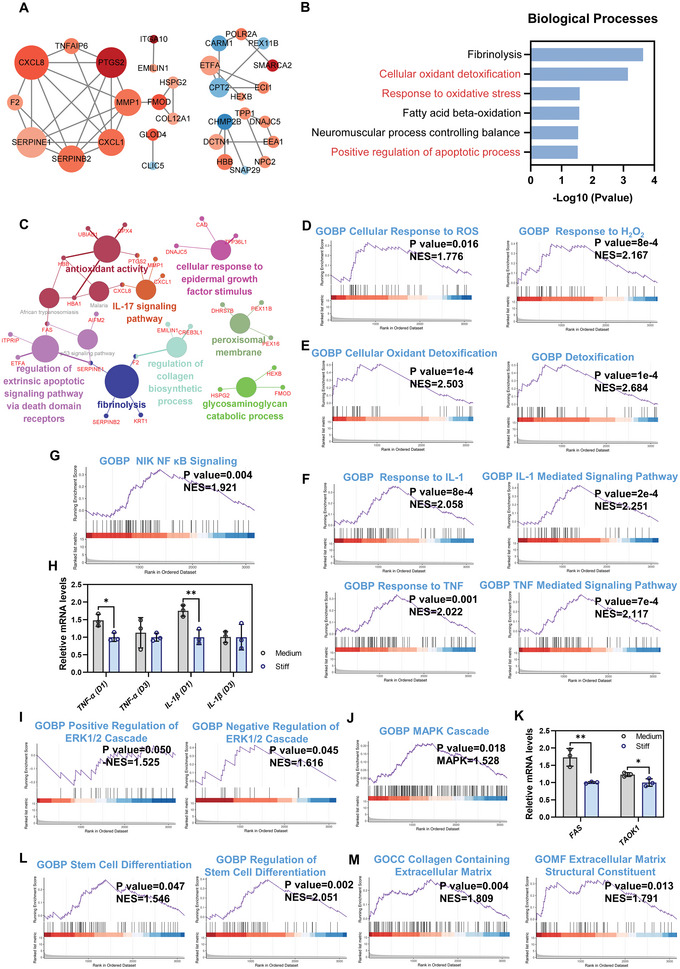
Functional changes of MSCs in Medium versus Stiff group under simultaneous stimulation of material stiffness and macrophage paracrine signals. A) Protein interaction network for the 30 hub DEPs in Medium versus Stiff. The larger size of the protein node indicated a higher frequency in the protein network. The up‐regulated proteins are presented in red while the down‐regulated are in blue. A deeper color of nodes represents a lower or higher fold change of DEPs. B) Key GO terms significantly enriched in BP (Medium versus Stiff). C) Network for GO and KEGG terms enriched from all DEPs (Medium versus Stiff). D) GSEA plot of cellular responses to ROS and H_2_O_2_ (Medium versus Stiff). E) GSEA plot of GO terms associated with antioxidant responses (Medium versus Stiff). F) GSEA plot of IL‐1 and TNF mediated signaling pathways (Medium versus Stiff). G) GSEA plot of NF‐*κ*B signaling pathway (Medium versus Stiff). H) *TNF‐α* and *IL‐1β* expression of MSCs treated with different stiffnesses and corresponding CMs on days 1 and 3 by qPCR (*n* = 3). I) GSEA plot of regulation of ERK1/2 signaling pathway (Medium versus Stiff). J) GSEA plot of MAPK signaling pathway (Medium versus Stiff). K) Gene expression of p38 MAPK signaling pathways related mediators on day 3 by qPCR (*n* = 3). L) GSEA plot of stem cell differentiation (Medium versus Stiff). M) GSEA plot of GO terms associated with collagen formation and organization (Medium versus Stiff). The results are shown as the mean ± SD. *p < 0.05; **p < 0.01 determined using two‐tailed Student's *t*‐test (H,K).

*Functional changes of MSCs in Medium versus Stiff group with simultaneous stimulation of stiffness and macrophage paracrine signals*: The protein interaction network for all DEPs when comparing the Medium and Stiff groups is shown in Figure S9A, Supporting Information. The 30 hub DEPs that could potentially play a critical role in the altered cellular processes and pathways were screened using CytoHubba in Cytoscape (**Figure** **5**A). It was observed that several inflammation‐related proteins, such as CXCL1, CXCL8, TNFAIP6, PTGS2 and MMP1, were significantly upregulated in the Medium group compared to the Stiff group, indicating that a strong inflammatory response could be activated in the Medium group (Figure 5A). To obtain further functional insights into the changes in protein expression, GO enrichment analysis was performed for all identified DEPs (Figure 5B, and Figure S9B,C, Supporting Information). The significant GO terms enriched in BP were fibrinolysis, cellular oxidant detoxification, response to oxidative stress, fatty acid beta‐oxidation, neuromuscular process controlling balance, and positive regulation of apoptotic process when comparing the Medium and Stiff groups (Figure 5B). We also observed that peroxidase activity and haptoglobin binding were significantly enriched in MF (Figure S9C, Supporting Information). For the changes in CC, only a few significant terms likely associated with tenogenic differentiation were observed, including extracellular exosome and extracellular matrix (Figure S9B, Supporting Information). In Figure 5C, a network for significant GO terms and KEGG pathways enriched from all DEPs by ClueGO in Cytoscape is illustrated. It was also observed that several cellular processes and pathways were associated with oxidative stress, inflammation, collagen biosynthesis and apoptosis, including antioxidant activity, IL‐17 signaling pathway, regulation of collagen biosynthetic process and regulation of extrinsic apoptotic signaling pathway via death domain receptors (Figure 5C). Collectively, our findings indicate that both oxidative stress and inflammatory responses were activated in the MSCs in the Medium group, with the inflammatory response being the most notable.

Subsequently, GSEA was performed to clarify the functional status of MSCs in the Medium versus the Stiff group. The results confirmed that some cellular responses associated with oxidative stress and antioxidant activities were activated, including cellular response to ROS, response to H_2_O_2_, antioxidant activity, oxidoreductase activity, cellular oxidant detoxification and detoxification (Figure 5D,E, and Figure S9D, Supporting Information). Collectively, our results confirmed that MSCs in the Medium group exhibited enhanced oxidative stress and antioxidant response compared to the Stiff group, which could be explained by the pro‐inflammatory properties of CM in the Medium group (Figure 2A–E, and Figure S7A,B, Supporting Information). In addition to oxidative stress, we found that the inflammatory response of MSCs in the Medium group was notably enhanced (Figure S9E, Supporting Information). Several typical pro‐inflammatory biological processes and signaling pathways were significantly activated in the Medium group, including response to IL‐1 and IL‐1 mediated signaling pathways, as well as response to TNF and TNF mediated signaling pathways, compared to the Stiff group (Figure 5F). The upregulated biological responses and signaling pathways in the Medium group could be explained by the increased IL‐1*β* and TNF‐*α* production by macrophages in response to medium stiffness (Figure 2C–E). Moreover, the NIK NF‐*κ*B signaling pathway, a classic pathway that induces pro‐inflammatory gene expression, was significantly activated in the Medium group as compared to the Stiff group (Figure 5G). Thus, we investigated the expression of pro‐inflammatory genes in MSCs after 1 and 3 d using qPCR (Figure 5H). The results revealed that *TNF‐α* and *IL‐1β* expression in the Medium group was significantly upregulated compared to that in the Stiff group on day 1 (Figure 5H). A previous study has demonstrated that a softer matrix results in inflammatory activation of MSCs and that simultaneous activation of pro‐inflammatory pathways, performed by enhancing the NF‐*κ*B signaling pathways, further induces the expression of downstream inflammatory genes, including *CCL2/7*, *IL6/8* and *TSG‐6*.^[^ ^29^ ^]^ Similarly, we found that paracrine signals from M1 macrophages cultured with Medium stiffness stimulated a pro‐inflammatory response with higher expression of TNF‐α and IL‐1β in MSCs (Figure 2C–E). When MSCs were cultured with Medium stiffness and simultaneously stimulated with CM from macrophages, further enhancement of inflammatory activation in MSCs was observed. The increase in inflammation was mediated via activation of the NF‐*κ*B signaling pathway, which resulted in significantly upregulated expression of inflammatory genes (*TNF‐α* and *IL‐1β*) and proteins (CXCL1, CXCL8, TNFAIP6, PTGS2, and MMP1) (Figure 5A,H). Thus, prominent inflammatory activation was induced in MSCs in the Medium group. A pro‐inflammatory environment with high levels of IL‐1*β* and TNF‐*α* has been shown to inhibit tenogenic differentiation and promote osteogenic differentiation of MSCs.^[^ ^26^, ^30^ ^]^ Thus, the combination of high levels of pro‐inflammatory factors (e.g., IL‐1*β* and TNF‐*α*) in the CM and the enhanced pro‐inflammatory expression of MSCs cultured on Medium stiffness inhibited the tenogenic differentiation of MSCs (Figure 2I–K). However, as shown in Figure 5I, inhibition of ERK1/2 signaling pathways seen at lower stiffness supported the tenogenic differentiation of MSCs (Figure 5I). Thus, the inhibition of ERK1/2 signaling partially explained why the promotion of tenogenic differentiation was still noticed when MSCs were cultured with pro‐inflammatory CM in the Medium group compared to the Stiff group (Figures 3B–D and 5L). Interestingly, although the ERK1/2 signaling pathway was significantly inhibited, the MAPK cascade was significantly activated in the Medium group compared to the Stiff group (Figure 5J). The MAPK cascade consists of four subfamilies: ERK1/2, P38 MAPK14 (stress‐activated MAPK cascade), JNK, and MEK cascades.^[^ ^31^ ^]^ Among these MAPK cascades, the P38 MAPK signaling cascade has been demonstrated to promote the expression of *SCX*, *TNMD*, and *COL1* in tenocytes.^[^ ^32^ ^]^ TAOK1 is an important activator of the p38 MAPK14 cascade and FAS has been demonstrated to be involved in P38 MAPK cascade activation by binding to the FAS ligand.^[^ ^33^ ^]^ Our results revealed that *TAOK1* and *FAS* in the Medium groups exhibited significantly increased expression compared to the Stiff group (Figure 5K), indicating that the p38 MAPK signaling cascade could be activated in the Medium group.^[^ ^34^ ^]^ Thus, the activated p38 MAPK signaling pathways could rescue and even reverse the inhibited tenogenic differentiation of MSCs by inflammatory factors in the Medium group, leading to the enhanced tenogenic potential of MSCs compared to that in the Stiff group (Figures 3B–D and 5L,M).

**Figure 6:**
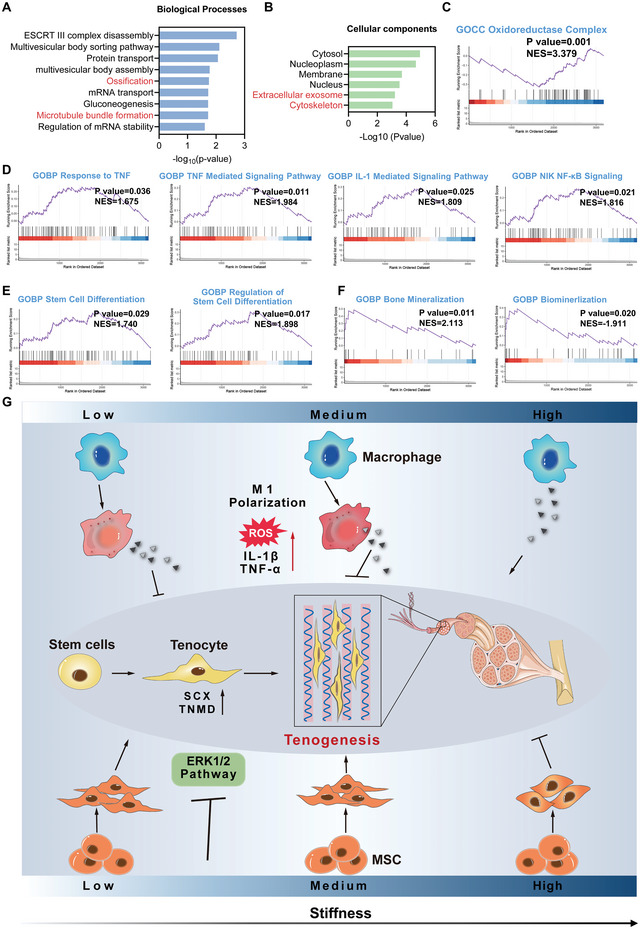
Functional changes of MSCs in Medium versus Soft group under simultaneous stimulation of material stiffness and macrophage paracrine signals. A,B) Key GO terms significantly enriched in BP and CC (Medium versus Soft). C) GSEA plot of oxidoreductase complex (Medium versus Soft). D) GSEA plot of inflammation‐related biological processes and signaling pathways (Medium versus Soft). E) GSEA plot of GO terms associated with stem cell differentiation (Medium versus Soft). F) GSEA plot of mineralization (Medium versus Soft). G) Schematic illustration of the influence of material stiffness and macrophage paracrine signals on the tenogenesis of stem cells.

*Functional changes of MSCs in Medium versus Soft group with simultaneous stimulation of stiffness and macrophage paracrine signals*: To clarify the difference in the functional status of MSCs between the Soft and Medium groups, we investigated changes in their cellular processes and pathways. GO enrichment analysis for BP, CC, and MF was conducted for all identified DEPs. The associated GO terms significantly enriched in BP, CC and MF were ossification, microtubule bundle formation, extracellular exosome, cytoskeleton and beta‐tubulin (**Figure** **6**A,B, and Figure S10A, Supporting Information). Notably, cytoskeleton‐related terms were significantly enriched in the Medium group compared to the Soft group, suggesting that MSCs in the two groups exhibited changes in their cytoskeletal organization and cell morphology. Importantly, ossification was significantly enriched in the Medium group compared to the Soft group, indicating that osteogenic differentiation of MSCs could be initiated (Figure 6A). GSEA was subsequently performed to clarify functional insights into MSCs between the groups. Our results revealed that the Medium group, compared to the Soft group, significantly promoted cell adhesion, actin filament formation and cytoskeleton organization, thereby regulating cell morphology, which could affect the lineage specification of MSCs (Figure S10D, Supporting Information). We observed that the oxidoreductase complex was suppressed in the Medium group compared to the Soft group (Figure 6C), which could be induced by its increased ROS production in MSCs (Figure S7A, Supporting Information). Due to the highest inflammation levels in the Medium group, several typical pro‐inflammatory cellular processes and signaling pathways were also detected, including the response to TNF, TNF‐mediated signaling pathway, IL‐1 mediated signaling pathway and NIK NF‐*κ*B signaling pathways (Figure 6D). The Medium group exhibited significantly upregulated stem cell differentiation compared to the Soft group (Figure 6E). However, it was observed that several biological processes associated with osteogenic differentiation were also significantly enriched in the Medium group compared to the Soft group, including enhanced bone mineralization and biomineralization, as well as inhibited bone resorption (Figure 6F, and Figure S10B, Supporting Information). qPCR further validated that the MSCs in the Medium group had significantly enhanced osteogenic differentiation with increased *RUNX2*, *OPN*, *ALP* and *OCN* expression compared to the Soft group, which could be explained by the increased stiffness and the higher levels of pro‐inflammatory factors in the Medium group compared to the Soft group (Figure S10C, Supporting Information).^[^ ^12^, ^30^ ^]^ In summary, MSCs in the Medium group exhibited slightly increased tenogenic gene expression and significantly enhanced osteogenesis compared to the Soft group. Considering the osteogenesis induced by the Medium group, it appeared that the Soft group was superior for tenogenic differentiation of MSCs when simultaneously stimulated by stiffness and macrophage paracrine signals (Figure 3B–D, and Figure S10C, Supporting Information).

In general, our results demonstrated that the effect of material stiffness on teno‐lineage differentiation of stem cells was stronger than that of paracrine signals from macrophages. The effect of CMs derived from macrophages cultured at different stiffnesses was opposite to the effect of stiffness itself on MSCs tenogenic differentiation. However, the positive role of lower stiffness covered or reversed the negative role of paracrine signals from macrophages. Thus, MSCs cultured on softer substrates showed enhanced tenogenic differentiation when simultaneously treated with macrophage CMs from the corresponding stiffnesses. Compared to the Stiff group, the CMs from M1 macrophages cultured on softer substrates promoted ROS production and subsequently activated the antioxidant responses of MSCs. It was difficult to clarify the influence of activated oxidative stress by CMs on the Soft and Medium groups when evaluating the tenogenic differentiation of MSCs because of their similar trend of ROS production and antioxidant responses. However, since the enhanced tenogenic differentiation on the softer substrates was accompanied by decreased expression of antioxidant enzymes (Figure S11, Supporting Information), we speculated that the oxidative stress induced by CMs in both Soft and Medium groups was not beneficial to tenogenic differentiation of MSCs. Compared to the CM from M1 macrophages cultured on the soft substrate, the CM collected from the medium substrate exhibited higher pro‐inflammatory properties. The CM with higher pro‐inflammatory properties could activate the typical pro‐inflammatory signaling pathways in MSCs, such as IL‐1*β*/TNF‐*α* and the NF‐*κ*B signaling pathway, which subsequently results in notable inflammatory activation. Interestingly, as the content of pro‐inflammatory cytokines in the CM increased in combination with significant inflammatory activation in MSCs, we suspected that the Medium group exhibited suppressed tenogenic differentiation compared to the Soft group. However, the opposite result was observed. This phenomenon could be attributed to the activation of the p38 MAPK signaling cascade due to higher pro‐inflammation in MSCs. Even though a slightly enhanced tenogenic differentiation of MSCs was found in the Medium group compared to the Soft group, the Medium group was not recommended due to its notably higher inflammation levels and enhanced osteogenic potentials. Moreover, we reasoned that the inflammation‐activated MSCs could further interact with M1‐polarized macrophages, which could result in a vicious circle that continuously promotes M1 phenotype polarization of macrophages, leading to chronic inflammation and the resultant impaired tendon healing.

### 2.4 Stiffness Regulates Macrophage Polarization and Ectopic Tendon Formation In Vivo

**Figure 7:**
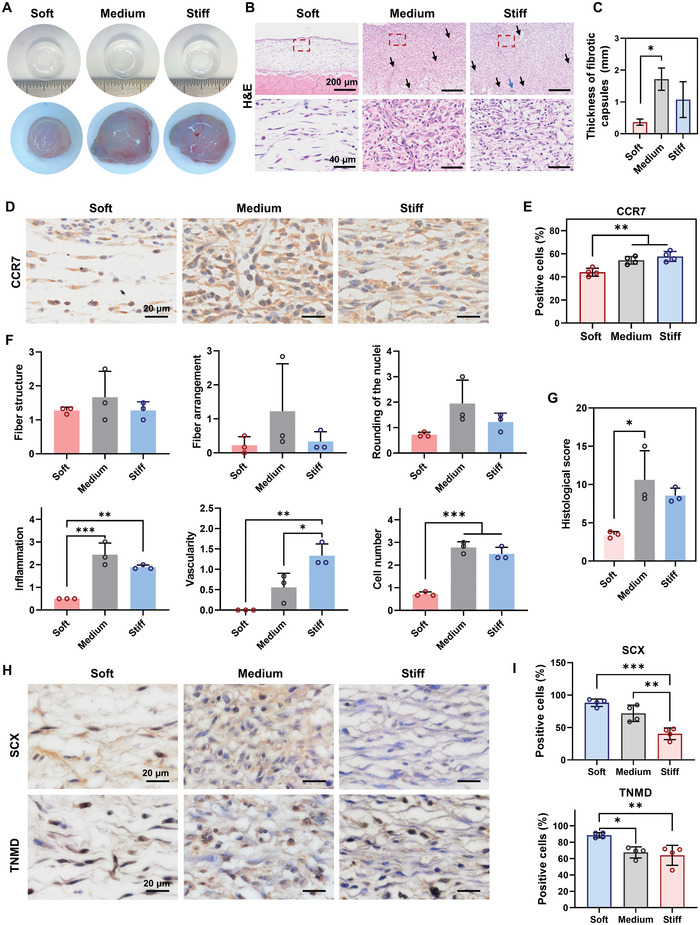
Material stiffness modulates macrophage polarization and ectopic tendon formation in vivo. A) Representative images of three PDMS substrates before and after in vivo implantation for 2 weeks. B) Representative images of H&E staining of samples in full thickness. Scale bars = 200 µm. The lower panel of images shows a high magnification of the fibrotic tissues marked in the red boxes of the corresponding picture in the upper panel. Scale bars = 40 µm. The samples were collected from the side of the PDMS where TDSCs were seeded. Blood vessels (black arrows) and FBGCs (blue arrows) were observed in the fibrotic tissues. C) Quantitative analysis of the thickness of the fibrotic capsule surrounding the PDMS (*n* = 3). D) Representative images of IHC staining for CCR7. Scale bars = 200 µm. E) Quantitative analysis of positive cells for CCR7 staining (*n* = 4). F) Histological scores for fiber structure, fiber arrangement, rounding of the nuclei, inflammation, vascularity, and cell number from H&E staining images (*n* = 3). G) Total histological scores. The tissue with a lower total score indicated a higher similarity to normal tendon tissue (*n* = 3). H) Representative images of IHC staining for SCX and TNMD. Scale bars = 20 µm. I) Quantitative analysis of positive cells for SCX and TNMD staining (*n* = 4). The results are shown as the mean ± SD. *p < 0.05; **p < 0.01; ***p < 0.001 determined using one‐way ANOVA with Tukey's post hoc test (C, E–G, and I).

To validate the host immune response to different stiffness conditions and evaluate the tendon‐inductive potential of material stiffness, PDMS with different stiffnesses was implanted subcutaneously in the dorsum of rats for 2 weeks. TDSCs were seeded on one side of the PDMS to create an environment for potential tendon tissue formation. **Figure** **7**A shows the gross observation of PDMS with different stiffnesses and the collagenous fibrotic capsules formed following in vivo implantation for 2 weeks. Both the Medium and Stiff groups exhibited larger and thicker collagenous fibrotic capsulation than did the Soft group, which was attributed to the activated inflammation or increased foreign body reaction in these two groups. Hematoxylin‐eosin (H&E) staining was performed to further evaluate the collagenous fibrotic tissues formed in the different groups. All samples for histological staining were collected from the side of the implanted PDMS where the TDSCs were seeded. As shown in Figure 7B, only the Soft group exhibited thin and sparse tissue formation, while both the Medium and Stiff groups had thick and dense fibrotic tissue formation. Moreover, quantitative analysis of H&E staining images confirmed that the Soft group had thinner fibrotic capsules than the other groups (Figure 7C). The thickest fibrotic capsule was observed in the Medium group (Figure 7C). Foreign body giant cells (FBGCs, blue arrow), formed by macrophage fusion, were observed in the Stiff group, but not in the other groups (Figure 7B). Moreover, immunohistochemical staining (IHC) for CCR7, a marker of M1 macrophages, was conducted to investigate macrophage polarization in the different groups. The staining images and quantitative analysis revealed that the percentage of CCR7 positive cells in the Medium and Stiff groups was notably higher than that in the Soft group, indicating that macrophages in response to higher stiffness were polarized into the M1 phenotype (Figure 7D,E). Taken together, medium stiffness triggered strong inflammatory activation after in vivo transplantation, which was consistent with our in vitro studies (Figures 2C–G and 5F–H). The increased pro‐inflammatory responses in the Medium group could be explained by the notably enhanced M1 polarization and the positive interaction between stem cells and macrophages. The Soft group showed a lower level of inflammation than did the Stiff group, which was not expected considering that the Soft group induced macrophage polarization into the M1 phenotype in vitro to a greater extent than did the Stiff group (Figure 2C–G). However, previous studies have demonstrated that other immune cells, especially neutrophils, can sense and interact with stiff materials in vivo and subsequently mediate pro‐inflammatory responses. The fact that other immune cells could be activated by the stiff material could explain why the Stiff group had significantly increased inflammation levels compared to the Soft group.^[^ ^35^ ^]^

To evaluate the tenogenic potential of materials with different stiffnesses in vivo, a quantified tendon histological score was obtained for the fiber structure, fiber arrangement, rounding of the nuclei, inflammation, vascularity and cell number (Figure 7F). We found that the Soft group exhibited significantly lower scores for inflammation, vascularity, and cell number than did the Medium and Stiff groups. The other scored parameters, including the fiber structure, fiber arrangement and rounding of the nuclei, showed a similar trend among the three groups. The total score of the Soft group (3.44 ± 0.42) was lower than that of the Medium (10.61 ± 3.81) and Stiff (8.56 ± 0.98) groups, suggesting that the tissues formed in the Soft group were more tendon‐like than those in the other groups (Figure 7G). IHC staining was conducted to evaluate the expression of SCX and TNMD in newly formed tissues after in vivo exposure to different stiffness levels. The percentage of SCX‐positive cells significantly increased as material stiffness decreased (Figure 7H,I). In addition, tissues in the Soft group showed a notably higher percentage of TNMD‐positive cells than those in the Medium and Stiff groups (Figure 7H,I). Taken together, these results show that the soft material promotes tendon‐like tissue formation in vivo, which could be explained by enhanced tenogenic differentiation of stem cells in a low inflammatory environment.

In this study, we investigated the influence of material stiffness on the cellular behavior of MSCs and the effect of the simultaneous stimulation of paracrine signals derived from macrophages. The main aim of this study was to determine the optimal material properties of the scaffolds for use in future implantation for tendon regeneration. To our knowledge, this is the first study to consider the effect of material stiffness and inflammatory responses of macrophages on tenogenic differentiation of stem cells. Our study provides comprehensive evidence for the optimized design and preparation of bioactive scaffolds for tendon tissue engineering. However, our study had some limitations. First, owing to the great potential of stem cells for tissue repair and regeneration,^[^ ^36^ ^]^ our study only focused on the influence of material stiffness and macrophage paracrine signals on the tenogenic differentiation of stem cells. However, the response of tenocytes, the primary cell population in tendons, to these factors has not been clarified. Second, macrophages are not the only immune cells that interact with biomaterials, and thus analyzing macrophages alone does not reflect the whole complexity of the inflammatory environment occurring in tendons in vivo. Third, PDMS with various stiffnesses provided a 2D model to clarify the influence of material stiffness on MSCs and macrophages. Future investigations based on a 3D model should be performed to further validate these cellular responses to material stiffness and their influence on tendon repair and regeneration.

## 3 Conclusion

In conclusion, we comprehensively compared the cellular responses of MSCs and macrophages, as well as their interactions via paracrine signaling, on PDMS substrates with different stiffnesses ranging from kilopascals to megapascals. Our data demonstrated that softer materials significantly stimulated the tenogenic differentiation of MSCs via the inhibition of ERK1/2 signaling pathways. However, the secretion of paracrine signals by M1 polarized macrophages cultured on soft and medium materials resulted in increased ROS production and enhanced oxidative stress in MSCs. Increased ROS production and enhanced oxidative stress in MSCs led to increased inflammatory activation, which inhibited the tenogenic differentiation of MSCs. When MSCs were cultured on materials with softer stiffnesses and simultaneously exposed to CMs derived from macrophages cultured at the corresponding stiffnesses, the MSCs still exhibited enhanced cell differentiation toward the tendon lineage. This indicates that the strong effect of material stiffness exceeds that of paracrine signals from macrophages on stem cells. In vivo, soft implanted materials induced low host inflammatory activation and promoted tendon‐like tissue formation. In general, soft biomaterials showed a greater potential to guide the differentiation of endogenous or exogenous stem cells into the tendon lineage and induce a more tendon‐like tissue formation as compared to stiffer ones. Our study provides comprehensive evidence for the optimized design and preparation of bioactive scaffolds in tendon tissue engineering and has the potential to be extended to a broader field of scaffold design and application.

## 4 Experimental Section

### Fabrication of PDMS Substrates with Different Stiffnesses

As previously reported, PDMS substrates were prepared with the commercially available Sylgard 184 silicone elastomer kit (Dow Corning, Midland).^[^ ^37^ ^]^ Different stiffnesses were performed by controlling the base and curing agents in the following ratios (w/w) (40:1 for soft; 25:1 for medium and 4:1 for stiff substrates). Subsequently, the well‐mixed prepolymer was poured into plastic culture plates. After incubation at 60 °C in a drying oven for 24 h, the PDMS substrates were sterilized with 75% ethanol and washed with PBS three times. Prior to cell cultivation, all the PDMS substrates employed in this study were coated with 0.1 mg mL^−1^ COL1 (CORNING, NY, USA) at 37 °C for 24 h to improve hydrophilicity and facilitate cell adhesion. The prepared PDMS substrates were stored at 4 °C before use.

### Characterization of PDMS Substrates with Different Stiffnesses

An electric universal testing machine (UTM2502 with a 50 N sensor; Sunstest, Shenzhen, China) was used to measure the compression modulus of PDMS substrates before and after COL1 coating and MSCs seeding. The samples with a diameter of 5 mm and a height of 3 mm were tested under compression at a strain rate of 4.8 mm min^−1^. With stress–strain curves generated by software, the modulus was calculated according to the slopes of the linear regions. The water contact angle of PDMS substrates before and after COL1 coating was determined using SA 100 surface analyzer (LAUDA Scientific, Lauda‐Königshofen, Germany) at room temperature and 50% humidity. A droplet of deionized water (10 µl) was dropped on the surface of PDMS substrates, and the image and the water contact value were collected when it was stable. The surface morphology of PDMS substrates before and after COL1 coating and MSCs seeding was observed by Zeiss EVO 18 SEM (Carl‐Zeiss, Oberkochen, Germany). The MSCs cultured on the PDMS substrates were fixed with 2.5% glutaraldehyde for 4 h and then dehydrated with ethanol in different concentrations ranging from 30% to 100% v/v. Subsequently, all PDMS substrates were dried inside a fume hood overnight and coated with gold for SEM analysis.

### Cell Culture

Immortalized human bone marrow MSCs (a gift from Prof. Zhou of Shandong Eye Institute, China) were cultured in DMEM‐low glucose medium (Wisent, Canada) supplemented with 10% fetal bovine serum (FBS, 086–550, Wisent, Canada) and 1% penicillin‐streptomycin (P/S, Gibco, Carlsbad, CA, USA) at 37 °C in 5% CO_2_ incubator. When performing experiments related to tenogenic differentiation, MSCs were cultured to confluency and then seeded into 6‐well plates or COL1‐coated PDMS substrates. The medium was switched to tenogenic medium, that is, DMEM‐high glucose medium (Gibco, Carlsbad, CA, USA) supplemented with 10% FBS, 1% P/S and 50 µg mL^−1^ L‐ascorbic acid 2‐phosphate (A2‐P; Sigma‐Aldrich, A8960).^[^ ^38^ ^]^

RAW 264.7 macrophages (murine mononuclear macrophage leukemia cells) utilized in this study were a gift from Prof. Wang of Southeast University, China. After expansion on tissue culture plastic (TCP, NEST Biotechnology, 704001), macrophages were seeded on COL1‐coated PDMS substrates with different stiffnesses and cultured in DMEM‐high glucose medium containing 10% FBS and 1% P/S at 37 °C in 5% CO_2_ incubator.

All cell experiments involving different stiffness conditions were performed on COL1‐coated PDMS substrates. For macrophage‐derived CMs preparation, the supernatant of the culture medium was collected and centrifuged at 2000 rpm for 20 min to remove the residual cells. The collected medium was filtered through 0.45 µm filters (Millipore, USA) and stored at −80 °C before use. A ratio of 5% FBS was added to CM to replenish the serum depleted by the culture of macrophages. When MSCs were cultured with CMs, 50 µg mL^−1^ A2‐P was supplemented for tenogenic differentiation.

### Cell Proliferation Assay

MSCs were seeded on PDMS substrates in a 96‐well plate. On days 1 and 3, the growth medium was replaced with a fresh medium supplemented with a 10% CCK‐8 reagent (APExBIO, Houston, TX). After incubation at 37 °C for 1 h in the dark, the test medium was collected and its OD value was measured using a microplate reader (BioTek, Winooski, VT, USA) at 450 nm.

### Cell Morphology

The morphology of MSCs exposed to different stiffnesses was examined with F‐actin staining. Cells were fixed in 4% v/v paraformaldehyde (PFA, Aladdin, China) and permeabilized with 0.1% Triton X‐100 (Beyotime, China). Actin Tracker Green (Beyotime, China) was diluted with PBS and added to plates to stain actin filaments. Nuclei were visualized by DAPI (Beyotime, China). Quantitative analysis for fluorescence intensity, cell spreading area, and cell aspect ratio was conducted in ImageJ software (NIH, Bethesda, MD).

### Gene Expression Analysis Using qPCR

At prescribed time‐points, total RNA extraction (Tiangen, China), reverse transcription (Toyobo, Japan), and qPCR analysis [Accurate Biotechnology (Hunan) Co., Ltd., AG11718] were carried out. Sequences of used primers (Genescript, Nanjing, China) are listed in Table S1, Supporting Information. Results were exhibited as a ratio of target gene expression to housekeeping gene *GAPDH*. The transcriptional expression stability of *GAPDH* had been evaluated in MSCs cultured on different PDMS substrates and tissue culture plastic (TCP) using *Bestkeeper* software, and it was confirmed that *GAPDH* was an appropriate reference gene for qPCR in the authors’ study.

### IF Staining

After 5 d of culture, MSCs were fixed with 4% PFA, permeabilized with 0.1% Triton X‐100, and then blocked with a blocking buffer for immunostaining (Beyotime, China) at room temperature. Subsequently, samples were incubated with rabbit anti‐SCX primary antibody (1:500, Abcam, China), anti‐TNMD primary antibody (1:200, Abcam, China), anti‐CD206 primary antibody (1:500, Proteintech, China), anti‐ARG‐1 primary antibody (1:250, Proteintech, China), and anti‐CCR7 primary antibody (1:250, Proteintech, China) overnight at 4 °C. On the following day, after three times washing with PBS, the samples were incubated with 488‐conjugated goat anti‐rabbit IgG (1:250, Proteintech, China) for 1 h in the dark at room temperature. The primary and secondary antibodies were diluted with NCM Universal Antibody Diluent (New Cell & Molecular Biotech, China). Nuclei were labeled using DAPI. The immunofluorescent intensity was quantified by ImageJ software.

### ROS Detections

Cellular ROS production under various conditions was measured with DCFH‐DA (Beyotime, China). DCFH‐DA was co‐incubated with cells in fresh serum‐free medium for 20 min at 37 °C. The excess probe was washed off thoroughly and then cells were observed under fluorescence microscopy (Carl‐Zeiss, Oberkochen, Germany). ImageJ software was applied to determine the intensity of fluorescence.

### Quantification of Cytokine Release (ELISA Assay)

ELISA Kit (Jingkang, China) was used to examine the IL‐1*β* production in macrophages under various conditions. CMs from macrophages were collected after 24 h of exposure to the PDMS substrates with different stiffnesses. The assay was performed according to the manufacturer's instructions. The optical density was determined using an 800 TS microplate reader at 450 nm immediately after adding the chromogenic agent. A standard concentration curve was constructed to calculate the exact concentration of each sample.

### Proteomic Analysis

After MSCs were treated with different stiffnesses and CMs for 3 d, cells were harvested for proteomic analysis. The LFQ‐based proteomic analysis was performed for global protein expressions as previously described.^[^ ^39^ ^]^ Proteomic data analysis was conducted in the DAVID, STRING, Cytoscape, CytoHubba, and the OmicStudio tools (https://www.omicstudio.cn/). Proteins with a *p*‐value less than 0.05 were identified as DEPs and employed for further enrichment analysis. The original proteomics data was uploaded to the iProX database (https://www.iprox.cn/, protein ID: IPX0004768000).

### In Vivo Subcutaneous Implantation Model

Animal experiments were performed according to the guidelines of the Animal Experimental Ethical Inspection Committee of Southeast University (No. 20220210060). PDMS substrates seeded with rat TDSCs were implanted subcutaneously into the dorsal section of the rats. Specifically, two symmetrical incisions were made on each rat's dorsum (left and right), and one substrate loaded with cells was implanted into each dorsal subcutaneous pocket. After the surgery, all the rats were fed with standard food and water and carefully monitored. At 2 weeks post‐surgery, the animals were sacrificed and implants were collected. After fixation in 4% v/v PFA for 48 h, samples were sectioned and stained with H&E and IHC staining for CCR7 (Proteintech, China), SCX (Abcam, China) and TNMD (Abcam, China). To evaluate the potential for tendon tissue formation, histological scores for fiber structure, fiber arrangement, nuclear roundness, vascularity, inflammation, and cell quantity were performed as previously described.^[^ ^40^ ^]^

### Statistical Analysis

All statistical analysis was performed using GraphPad Prism software. The unpaired two‐tailed student's *t*‐test (for two groups) and one‐way ANOVA followed by a Tukey post‐hoc test (for more than two groups) were used for statistical analysis. Statistically significant differences were recognized at *p* < 0.05 and were presented as ns (*p* > 0.05), * (*p* < 0.05), **(*p* < 0.01), and *** (*p* < 0.001). The results were presented as mean ± standard deviation (SD). In vitro experiments were performed in triplicates or more per experimental group (*n* ≥ 3).

## Conflict of Interest

The authors declare no conflict of interest.

## Supporting information

Supporting Information

## Data Availability

The data that support the findings of this study are available from the corresponding author upon reasonable request.
