# Supplementary material for: Material Stiffness in Cooperation with Macrophage Paracrine Signals Determines the Tenogenic Differentiation of Mesenchymal Stem Cells
Source: Adv Sci (Weinh). 2023 Apr 25;10(17):2206814. doi: 10.1002/advs.202206814 (PMC10265049; doi:10.1002/advs.202206814)
Supplement: Supplementary file 1 — Supporting Information [file ADVS-10-2206814-s001.pdf]

ADVANCED SCIENCE
Open Access

# Supporting Information

for *Adv. Sci.*, DOI 10.1002/advs.202206814

Material Stiffness in Cooperation with Macrophage Paracrine Signals Determines the Tenogenic Differentiation of Mesenchymal Stem Cells

*Renwang Sheng, Jia Liu, Wei Zhang, Yifan Luo, Zhixuan Chen, Jiayu Chi, Qingyun Mo, Mingyue Wang, Yuzhi Sun, Chuanquan Liu, Yanan Zhang, Yue Zhu, Baian Kuang, Chunguang Yan, Haoyang Liu, Ludvig J. Backman and Jialin Chen**

ADVANCED SCIENCE
Open Access

Supporting Information

for *Adv. Sci.*, DOI 10.1002/advs.202206814

Material Stiffness in Cooperation with Macrophage Paracrine Signals Determines the Tenogenic Differentiation of Mesenchymal Stem Cells

*Renwang Sheng, Jia Liu, Wei Zhang, Yifan Luo, Zhixuan Chen, Jiayu Chi, Qingyun Mo, Mingyue Wang, Yuzhi Sun, Chuanquan Liu, Yanan Zhang, Yue Zhu, Baian Kuang, Chunguang Yan, Haoyang Liu, Ludvig J. Backman and Jialin Chen**

# Supporting Information

**Material Stiffness in Cooperation with Macrophage Paracrine Signals Determines the Tenogenic Differentiation of Mesenchymal Stem Cells**

*Renwang Sheng[1], Jia Liu[1], Wei Zhang[1], Yifan Luo, Zhixuan Chen, Jiayu Chi, Qingyun Mo, Mingyue Wang, Yuzhi Sun, Chuanquan Liu, Yanan Zhang, Yue Zhu, Baian Kuang, Chunguang Yan, Haoyang Liu, Ludvig J. Backman, Jialin Chen**

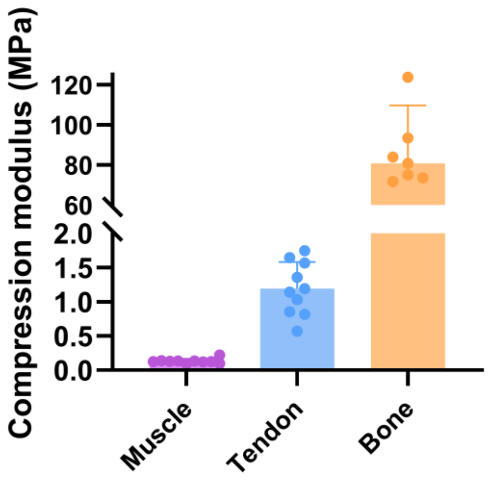


**Figure S1. Compressive modulus of the muscle (soleus), tendon (Achilles' tendon), and bone (tibia) of SD rat.** The results are shown in mean ± SD.

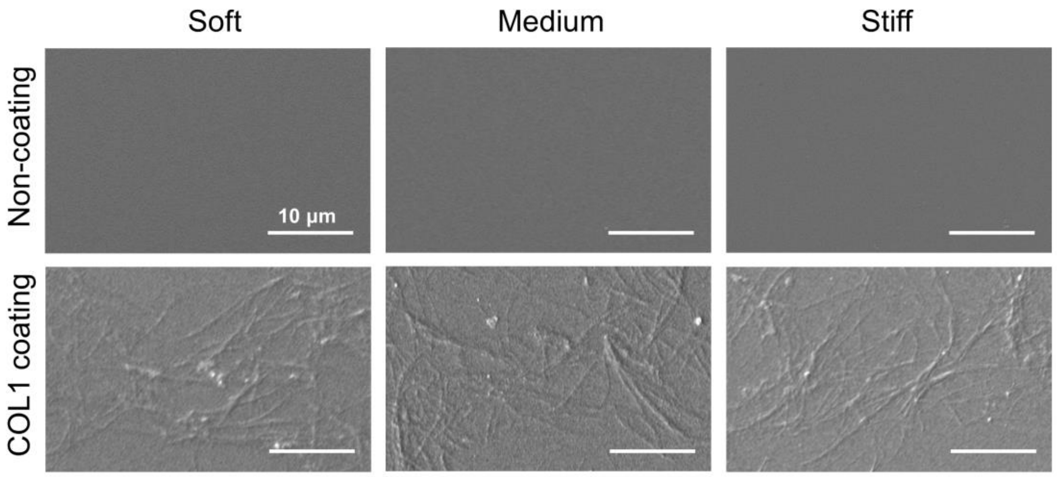


**Figure S2. SEM images of three PDMS substrates before and after COL1 coating.** Scale bar = 10 μm.

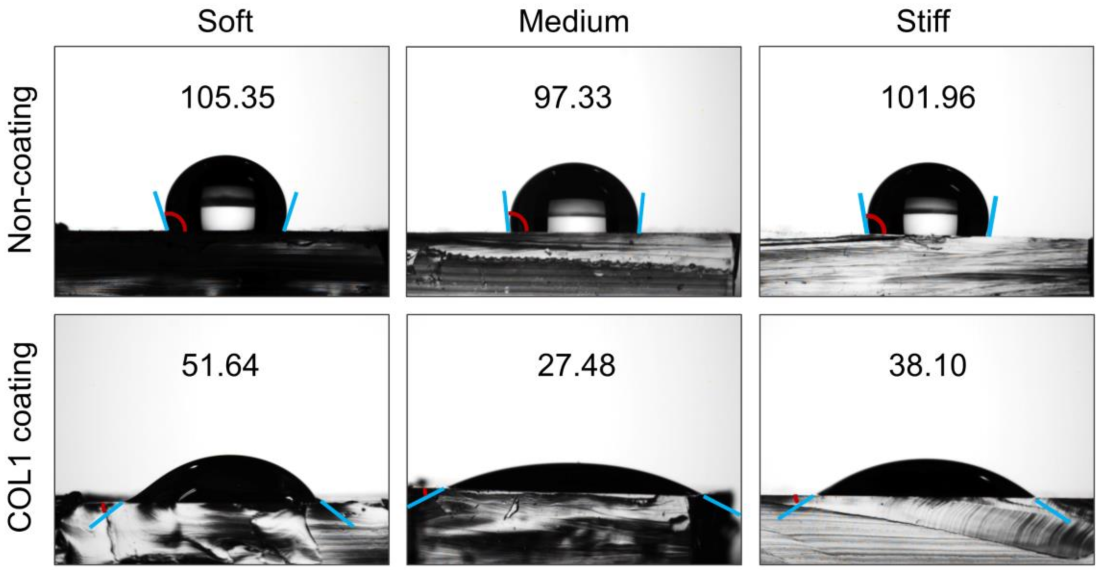


**Figure S3. Water contact angles of three PDMS substrates before and after COL1 coating.**

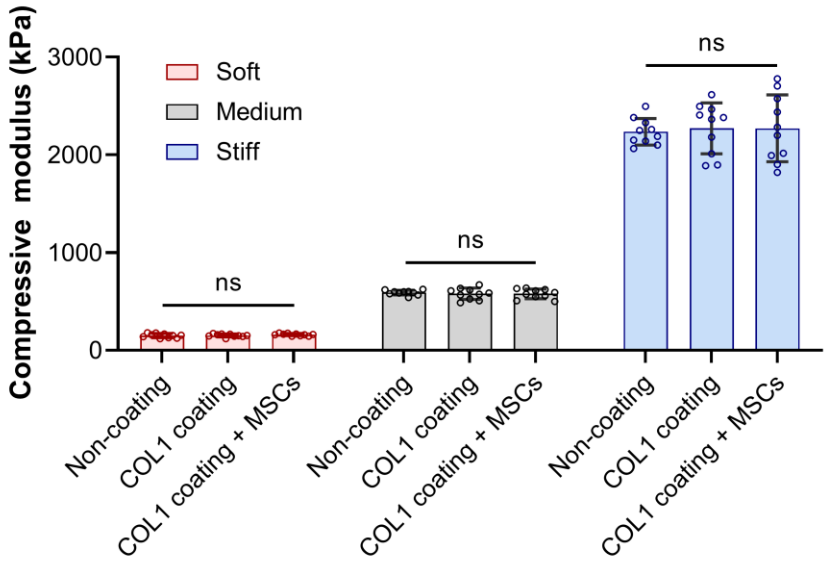


**Figure S4. Compressive modulus of three PDMS substrates before and after COL1 coating and MSCs seeding (n = 10).** The results are shown in mean ± SD. ns (P > 0.05) determined using one-way ANOVA with Tukey's post hoc test.

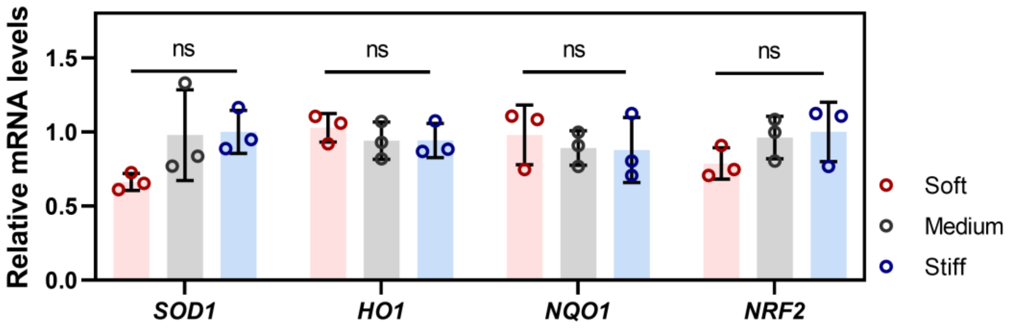


**Figure S5. Gene expression of antioxidant mediators in macrophages on day 3 by qPCR (n = 3).** The results were shown in mean ± SD. ns (P > 0.05) determined using one-way ANOVA with Tukey's post hoc test.

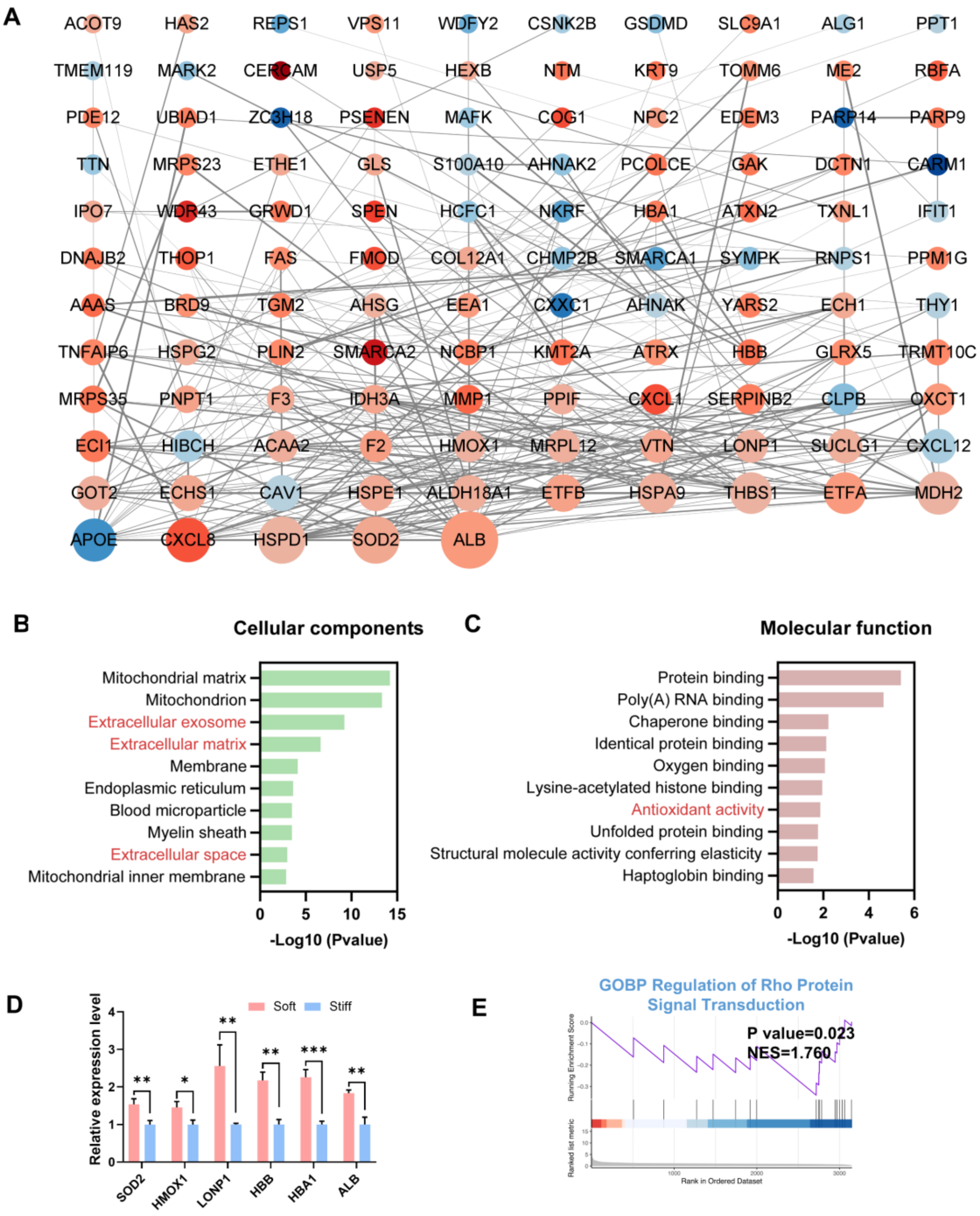


**Figure S6. Functional insights of DEPs in Soft vs. Stiff.** (A) The protein interaction network for all DEPs (Soft vs. Stiff). The larger size of the protein node indicates a higher frequency in the protein network. The up-regulated proteins are presented in red while the down-regulated are in blue. A deeper color of nodes represents a lower or higher fold change of DEPs. (B-C) Key GO terms significantly enriched in CC and MF (Soft vs. Stiff). (D) Protein expression of several antioxidative mediators from proteomic profiles (n = 3). (E) GSEA plot of regulation of Rho protein signal transduction (Soft vs. Stiff). The results are shown in mean ± SD. *p < 0 .05; **p < 0.01; ***p < 0.001 determined using two-tailed student's t-test (D).

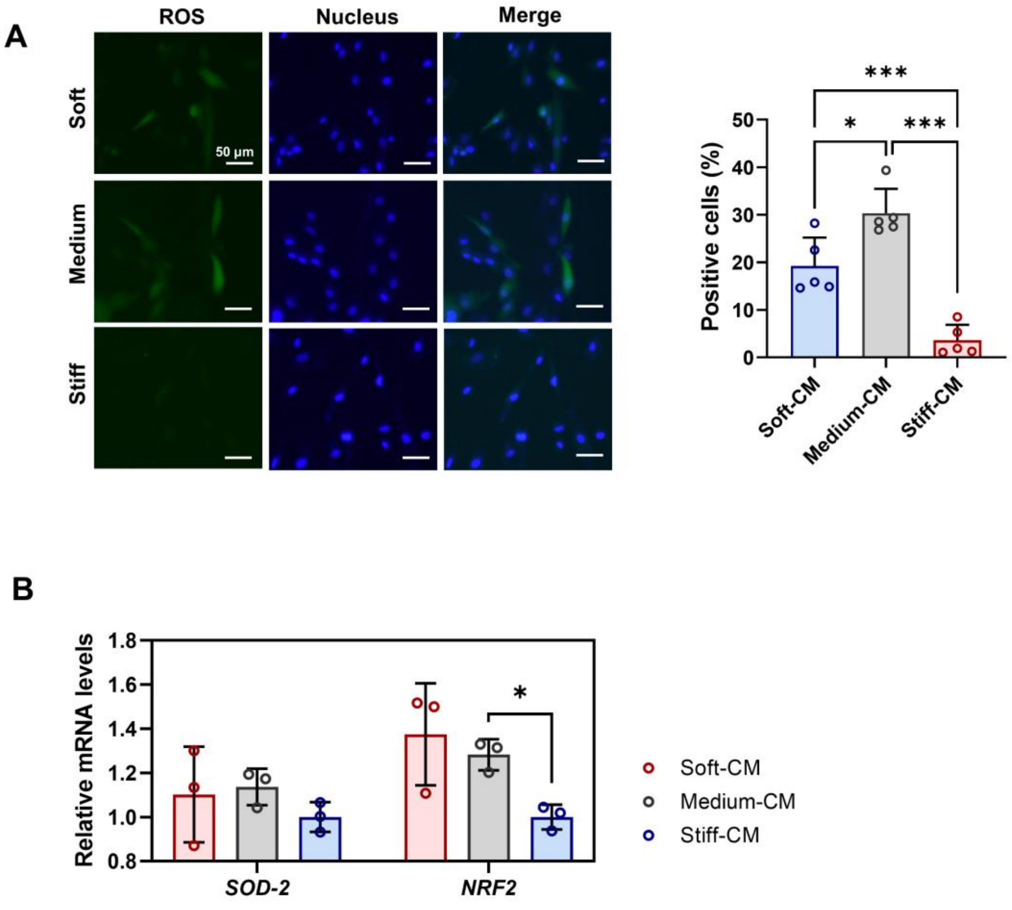


**Figure S7. Influences of different CMs on the oxidative stress of MSCs.** (A) Representative images and quantitative analysis of ROS staining in MSCs exposed to different CMs for 1 d (n = 5). Scale bar = 50 μm. (B) Gene expression of *SOD2* and *NRF2* in MSCs exposed to different CMs by qPCR (n = 3). The results are shown in mean ± SD. *$p < 0.05$; **$p < 0.01$; ***$p < 0.001$ determined using one-way ANOVA with Tukey's post hoc test.

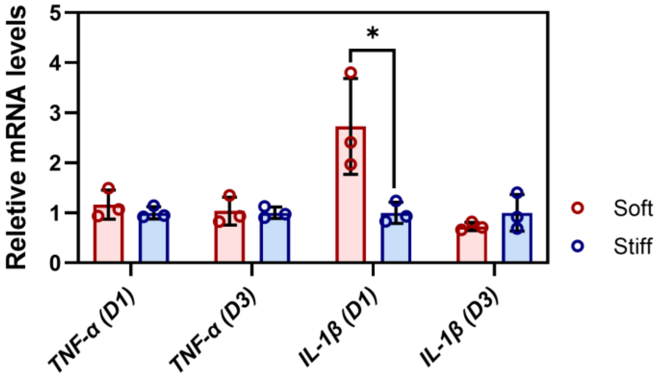


**Figure S8. Gene expression of *TNF-α* and *IL-1β* in MSCs exposed to different stiffnesses and CMs on days 1 and 3 by qPCR (n = 3).** The results are shown in mean ± SD. *$p < 0.05$ determined using two-tailed student's t-test.

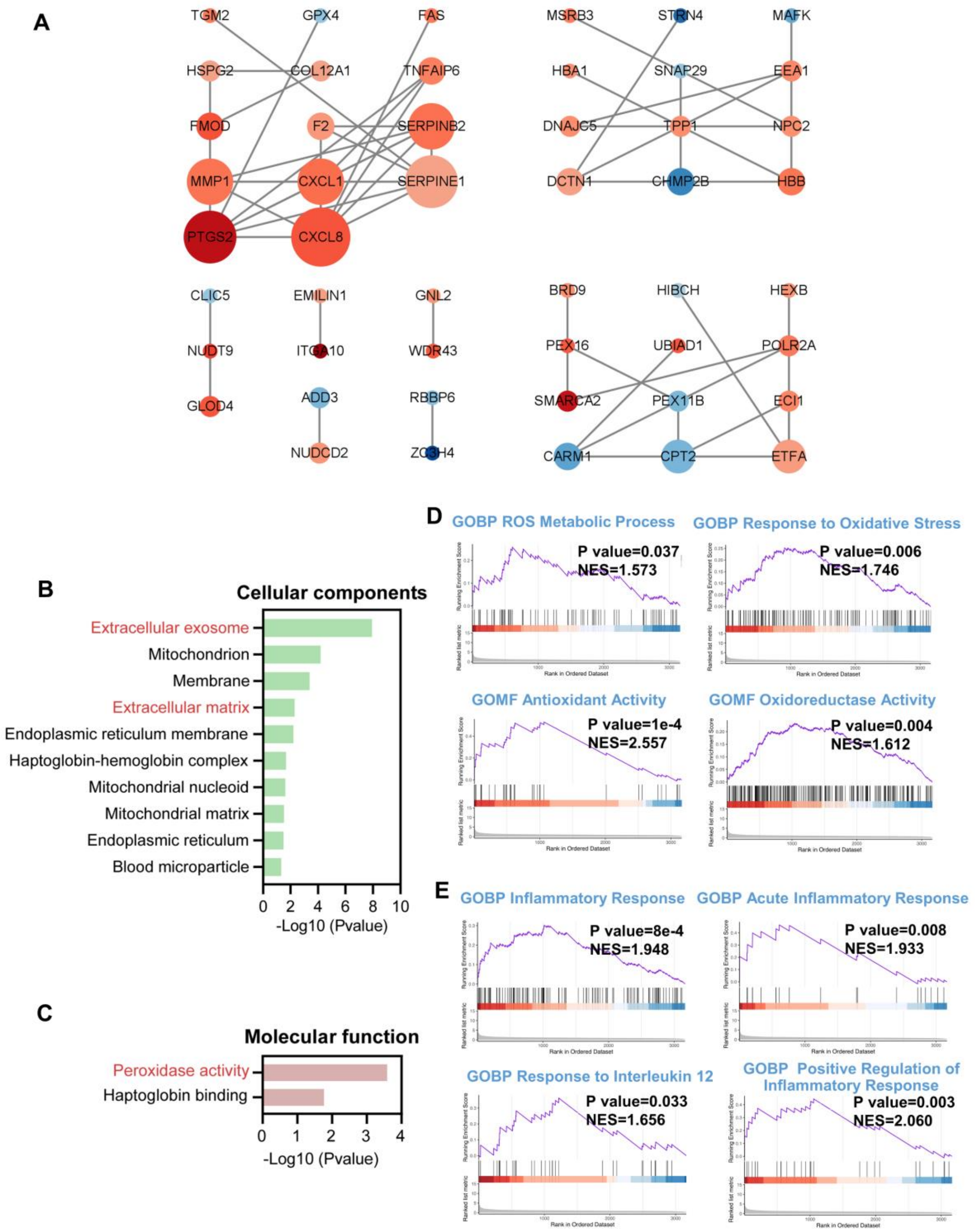


**Figure S9. Functional insights of DEPs in Medium vs. Stiff.** (A) The protein interaction network for all DEPs (Medium vs. Stiff). The larger size of the protein node indicated a higher frequency in the protein network. The up-regulated proteins are presented in red while the down-regulated are in blue. A deeper color of nodes represents a lower or higher fold change of DEPs. (B-C) Key GO terms significantly enriched in CC and MF (Medium vs. Stiff). (D) GSEA plot of GO terms associated with oxidative stress (Medium vs. Stiff). (E) GSEA plot of GO

terms associated with inflammation (Medium vs. Stiff).

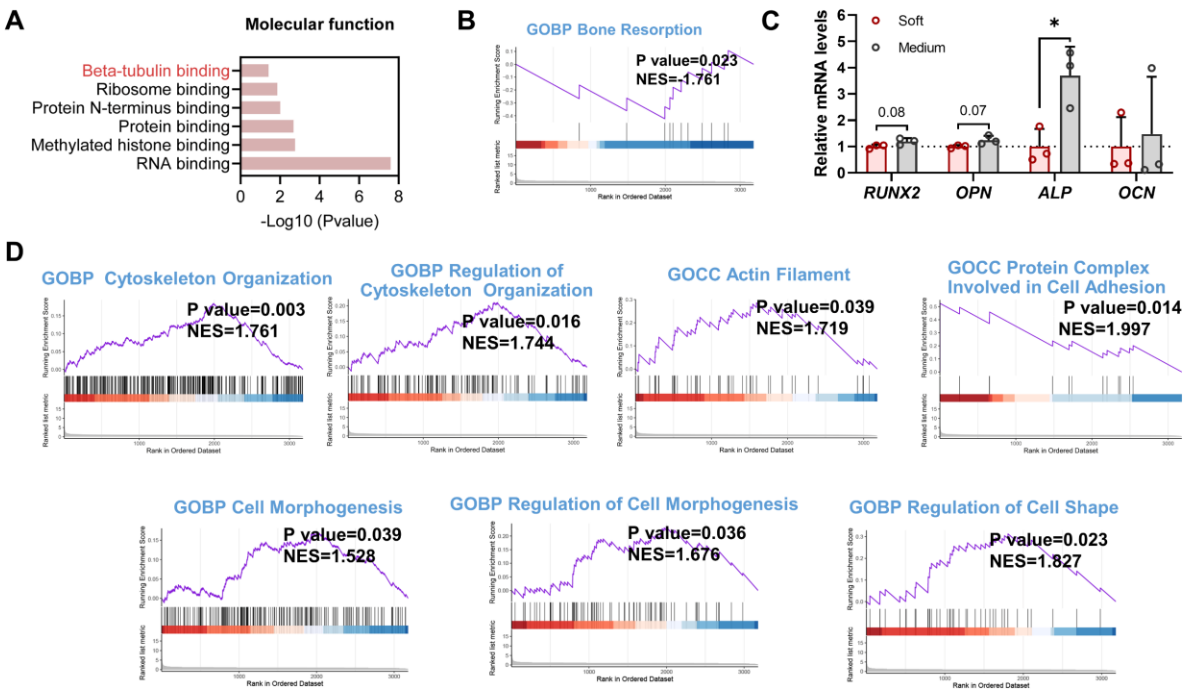


**Figure S10. Functional insights of DEPs in Medium vs. Soft.** (A) Key GO terms significantly enriched in MF (Medium vs. Soft). (B) GSEA plot of bone resorption (Medium vs. Soft). (C) Gene expression of osteogenic markers in MSCs on day 1 by qPCR (n = 3). (D) GSEA plot of GO terms associated with focal adhesion, cytoskeletal organization, and cell morphogenesis (Medium vs. Soft). The results are shown in mean ± SD. *p < 0.05 determined using two-tailed student's t-test (C).

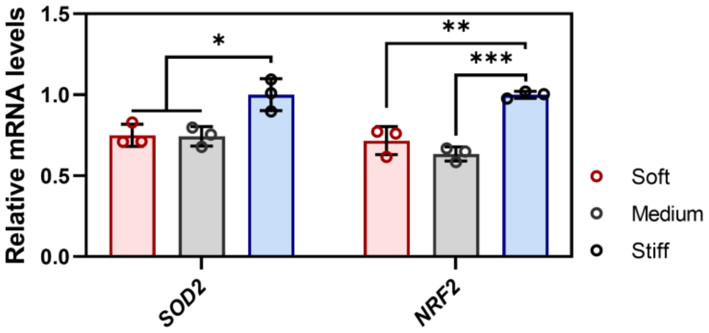

**Figure S11. Gene expression of *SOD2* and *NRF2* in MSCs cultured on different substrates on day 3 by qPCR (n = 3).** The results are shown in mean ± SD. $*p < 0.05$; $**p < 0.01$; $***p < 0.001$ determined using one-way ANOVA with Tukey's post hoc test.

**Table S1. Primer sequences are specific to MSCs and Raw264.7 for qPCR.**

| Species | Gene | Forward & Reverse | Sequence (5'- 3') |
|---|---|---|---|
| Human | *GAPDH* | Forward | TGACGCTGGGGCTGGCATTG |
| | | Reverse | GGCTGGTGGTCCAGGGGTCT |
| Human | *SCX* | Forward | CGAGAACACCCAGCCCAAAC |
| | | Reverse | CTCCGAATCGCAGTCTTTCTGTC |
| Human | *MKX* | Forward | GAAGGCAACTTTGTCTATCGCA |
| | | Reverse | TGATCTCCTTCCAATACGTGTC |
| Human | *TNMD* | Forward | CCATGCTGGATGAGAGAGGT |
| | | Reverse | CTCGTCCTCCTTGGTAGCAG |
| Human | *COL1* | Forward | CGATGGATTCCAGTTCGAGTAT |
| | | Reverse | CATCGACAGTGACGCTGTAGG |
| Human | *COL3* | Forward | TTTTGCAGTGATATGTGATGTT |
| | | Reverse | GGATGGTGGTTTTCAGTTTA |
| Human | *COL14* | Forward | AAGGATTGCCCTCCGACTACAC |
| | | Reverse | CTGATGCGTTCATTGCCTTCTC |
| Human | *SOD2* | Forward | CTGGACAAACCTCAGCCCTA |
| | | Reverse | TGATGGCTTCCAGCAACTC |
| Human | *NRF2* | Forward | AGTGGATCTGCCAACTACTC |
| | | Reverse | CATCTACAAACGGGAATGTCTG |
| Human | *IL-1β* | Forward | GCAAGGGCTTCAGGCAGGCCGCG |
| | | Reverse | GGTCATTCTCCTGGAAGGTCTGTGGGC |
| Human | *TNF-α* | Forward | ATGTTGTAGCAAACCCTCAAGC |
| | | Reverse | TGATGGCAGAGAGGAGGTTG |
| Human | *MAP2K2* | Forward | CGGACCTGAAGATGCTCACA |
| | | Reverse | TTCAGCCGCAGGGTTTTACA |

| | | | |
|---|---|---|---|
| Human | *MAP4K4* | Forward | GGGGAACGCTTCAGAGTGAG |
| | | Reverse | GTGCGGTCAGATCAGCAGG |
| Human | *TAOK1* | Forward | CCTCCTCCTCCTCACTCCTC |
| | | Reverse | GGCATAAACTACCTTTCGCCG |
| Human | *FAS* | Forward | ATTATCGTCCAAAAGTGTTA |
| | | Reverse | TCACACAATCTACATCTT CTG |
| Mouse | *GAPDH* | Forward | GCAAGTTCAACGGCACAG |
| | | Reverse | CGCCAGTAGACTCCACGAC |
| Mouse | *CCR7* | Forward | ATGGACCCAGGTGTGCTTCT |
| | | Reverse | TCAGTATCACCAGCCCGTTG |
| Mouse | *iNOS* | Forward | CCTGTGTTCCACCAGGAGAT |
| | | Reverse | CCCTGGCTAGTGCTTCAGAC |
| Mouse | *IL-1β* | Forward | AAGGAGAACCAAGCAACGACAAAA |
| | | Reverse | TGGGGAACTCTGCAGACTCAAACT |
| Mouse | *TNF-α* | Forward | CGTCAGCCGATTTGCTATCT |
| | | Reverse | CGGACTCCGCAAAGTCTAAG |
| Mouse | *IL-10* | Forward | CCAAGCCTTATCGGAAATGA |
| | | Reverse | TTTTCACAGGGGAGAAATCG |
| Mouse | *CD206* | Forward | AGCTTCATCTTCGGGCCTTTG |
| | | Reverse | GGTGACCACTCCTGCTGCTTTAG |
| Mouse | *SOD1* | Forward | AACCAGTTGTGTTGTCAGGAC |
| | | Reverse | CCACCATGTTTCTTAGAGTGAGG |
| Mouse | *HO1* | Forward | GATAGAGCGCAACAAGCAGAA |
| | | Reverse | CAGTGAGGCCCATACCAGAAG |
| Mouse | *NQO1* | Forward | AGGATGGGAGGTACTCGAATC |
| | | Reverse | TGCTAGAGATGACTCGGAAGG |
| Mouse | *NRF2* | Forward | TAGATGACCATGAGTCGCTTGC |
| | | Reverse | GCCAAACTTGCTCCATGTCC |
